# Grape ripening heterogeneity in white and red cultivars ( *Vitis vinifera* L.) grown on different calcareous soils using technological and phenolic markers

**DOI:** 10.1002/jsfa.70231

**Published:** 2025-10-08

**Authors:** Clara Vitaggio, Matteo Pollon, Manuel Schnitter, Valentina Caraci, Luciano Cinquanta, Onofrio Corona

**Affiliations:** ^1^ Department of Agricultural, Food and Forest Sciences University of Palermo Palermo Italy

**Keywords:** grape variability, grape maturity, density sorting, wine quality, phenolic compounds, winegrapes

## Abstract

**BACKGROUND:**

An understanding of grape ripening heterogeneity is essential to optimize vineyard management and to ensure wine quality. This study investigated the effect of calcareous soils with different textures (limestone and marlstone) on the chemical composition of white (‘Grillo’ (GR) and ‘Chardonnay’ (CH)) and red (‘Nero d’Avola’ (ND) and ‘Syrah’ (SY)) grape cultivars, including both autochthonous and allochthonous types. Grape quality was evaluated in relation to soil type using key compositional parameters and the degree of ripening homogeneity at harvest, quantified with two novel indices: the technological variability index (TVI) and the phenolic variability index (PVI).

**RESULTS:**

Soil type was the dominant factor influencing grape physicochemical and phenolic composition. Grapes grown on marlstone soils showed more homogeneous ripening, indicated by lower TVI and PVI values, in comparison with those grown on limestone. Autochthonous varieties exhibited greater adaptability and ripening uniformity across soil types than international cultivars. Multivariate analysis further revealed distinct soil–cultivar interactions for red and white grapes, underscoring genotype‐dependent responses to soil properties.

**CONCLUSION:**

Grape ripening behavior is influenced strongly by both cultivar and soil texture. Autochthonous cultivars, particularly GR and ND, exhibited greater ripening uniformity at harvest, suggesting a stronger adaptation to their native terroirs. Marlstone soils consistently promoted more homogeneous ripening, likely due to their superior water retention capacity. These findings highlight the importance of understanding soil effects in optimizing grape quality and support the development of more effective cultivar–soil matching strategies, especially in the face of increasing climate variability. © 2025 The Author(s). *Journal of the Science of Food and Agriculture* published by John Wiley & Sons Ltd on behalf of Society of Chemical Industry.

**ABBREVIATIONS**
CH: ‘Chardonnay’
GR: ‘Grillo’
LIM: limestone
MAR: marlstone
ND: ‘Nero d'Avola’
SY: ‘Syrah’

## INTRODUCTION

The concept of terroir refers to the multifactorial interaction between environmental conditions, soil characteristics, and the genotypic traits of the cultivar. This interaction, further influenced by agronomic and enological practices, plays a crucial role in shaping the technological and sensory attributes of wine.^1^ Prestigious wine‐growing regions characterized by calcareous soils, such as Burgundy, Champagne, Langa, Marlborough, and parts of Sicily, are particularly well suited to producing high‐quality wines.^1^

Calcareous soils typically exhibit low levels of organic matter and a reduced cation exchange capacity – characteristics closely linked to soil texture. Soil texture fundamentally influences key soil–plant interactions, particularly those governing water dynamics and their effects on grapevine physiology. Calcareous soils are frequently linked to improved grape quality, as the limited availability of essential nutrients, such as phosphorus, iron, and zinc, in those soils tends to shift the vine's resource allocation from vegetative growth toward reproductive development.^1^, ^2^, ^3^

Achieving optimal grape maturity in the vineyard is essential for producing high‐quality wines, particularly regarding sugar accumulation, acidity, flavor development, and polyphenolic evolution, including tannin maturation and anthocyanin accumulation in red grapes. Grape ripening is traditionally assessed through vineyard sampling to determine technological maturity, defined as the stage at which pulp sugar accumulation is maximal, acidity is low, and the sugar‐to‐acidity ratio is high.^4^ However, this assessment generally provides only an average estimate of grape ripening status and does not adequately account for heterogeneity within the vineyard.

Grape ripening is inherently heterogeneous, influenced by vineyard location, the berry position within the cluster, cluster position in the vine, and berry size.^4^ This variability is evident from the onset of veraison (where noticeable differences in berry coloration within the same cluster indicate varying stages of maturity) and it decreases as ripening progresses.^5^ During ripening, growth‐related genes in less advanced berries are overexpressed, allowing them to catch up and reduce the developmental gap observed at veraison.^6^, ^7^ However, if the plant cannot maintain photosynthetic activity under adverse conditions, such as heatwaves and prolonged drought, it may fail to compensate for these gaps, leading to inhomogeneous ripening.

This phenomenon is increasingly common in warm and arid regions as a consequence of climate change, where prolonged periods of high temperature and water stress prevail. Temperatures above 35 °C can disrupt vine physiology and berry development, whereas extreme events above 40–45 °C may impair photosynthesis, cause tissue damage, and reduce yields.^8^ Beyond yield reduction, heat stress also alters berry composition by delaying ripening when occurring at green stages or by accelerating sugar accumulation while limiting anthocyanin and acid retention during ripening, ultimately promoting heterogeneous maturity within vineyard parcels.^9^

This phenomenon can lead to the co‐existence of berries with markedly different metabolite composition within the same harvest, which can negatively affect wine sensory and chemical characteristics,^10^, ^11^ creating technical challenges for the winemaker. Unripe grapes are generally characterized by low sugar content, high acidity, low concentration of anthocyanins, and high concentration of flavonoids (especially from seeds).^12^ Their presence produces sensory notes of greenness or unripe fruit in the final wine.^13^ The unripe character is perceived as a puckering sensation from the mid‐palate through the aftertaste, often mistakenly attributed to high acidity.^13^ Such wines are also deficient in volatile compounds, which are synthesized in grape berries as they approach full ripeness.^14^

In warm‐arid regions, growers increasingly harvest earlier to limit excessive sugar accumulation and preserve freshness in wines.^14^ This approach effectively controls alcohol levels but it may increase the proportion of underripe berries within the harvest. Conversely, overripe grapes tend to produce wines with excessively high alcohol concentrations, port‐like aromas, and a sensation of hotness in the palate.^15^ Managing grape heterogeneity can therefore be considered a strategy to produce full‐flavored wines with moderate ethanol concentrations.

Uneven ripening is influenced primarily by soil type and depth, as these geophysical parameters are closely associated with vine vigor and yield, which in turn influence berry composition and wine quality.^16^ In‐field grape variability, driven by physical and environmental factors such as soil, topography, and climate has encouraged the adoption of precision viticulture strategies, including staggered harvesting.^17^

Grape heterogeneity represents a complex and challenging viticultural issue, not only in terms of control but also in terms of objective quantification. Measurements derived from representative vineyard sampling and replicate analyses provide reliable estimates of the mean ripening level of a vineyard parcel but cannot capture the degree of heterogeneity in berry maturation and may lead to suboptimal decisions regarding the harvest date.

Armstrong *et al*.^18^ developed a grape heterogeneity index (GHI) as a novel metric to assess overall grape heterogeneity. The GHI was calculated as the sum of absolute residuals of seven grape maturity parameters, including total soluble solids, pH, berry fresh weight, malic acid, 3‐isobutyl‐2‐methoxypyrazine, total tannins, and absorbance at 520 nm, multiplied by the range of values at the bunch level, and applied on multiple dates during the two vintages to assess the effects of viticultural treatments on overall grape heterogeneity. The idea proposed in their work was that aggregating multiple measures of grape maturity variability into a composite index would improve data summarization and facilitate straightforward comparisons. However, because the GHI is relative to the dataset considered, it remains difficult to compare across different datasets or historical data.

Given the importance of capturing grape ripening heterogeneity for optimizing harvest timing, ensuring wine quality, and identifying the most suitable grape variety for a given soil type, this study aimed to develop variability indices that can be readily compared with each other and with historical data. In this context, a technological parameters variability index and a phenolic variability index were constructed, each incorporating key technological and phenolic parameters considered most relevant from an enological standpoint. These indices were applied to both traditional grape varieties cultivated in Sicily (‘Grillo’ (GR) and ‘Nero d’Avola’ (ND)) and international grape varieties, including two white cultivars (‘Grillo’ (GR) and ‘Chardonnay’ (CH)) and two red cultivars (‘Nero d’Avola’ (ND) and ‘Syrah’ (SY)), grown on calcareous soils with different textures, limestone and marlstone, across two vintages.

## MATERIALS AND METHODS

### Vineyards

The research took place during the 2021 and 2022 vintages in four commercial vineyards situated in the hills near Menfi (AG), along the southwestern coastline of Sicily, Southern Italy.

‘Chardonnay’, GR, ND, and SY were manually harvested from limestone soils (LIM) and marlstone soils (MAR). The CH limestone vineyard (CH LIM) was located at 37° 38′ 31.0″ N, 12° 56′ 48.1″ E, and the CH marlstone vineyard (CH MAR) at 37° 38′ 44.6″ N, 12° 58′ 23.8″ E. The GR limestone vineyard (GR LIM) was located at 37° 35′ 27.1″ N, 13° 00′ 45.4″ E, and the GR marlstone vineyard (GR MAR) at 37° 38′ 42.2″ N, 12° 57′ 59.9″ E (Supporting Information, Figs S1 and S2).

The CH LIM soil was composed of 61% sand, 31% clay, and 8% silt; CH MAR was composed of 35% sand, 36% clay, and 29% silt; GR LIM was composed of 59% sand, 21% clay, and 20% silt; GR MAR was composed of 18% sand, 51% clay, and 31% silt (Supporting Information, Tables S1–S4).

‘Nero d’Avola’ from the limestone vineyard (ND LIM) was located at 37° 38′ 56.6″ N, 12° 59′ 45.6″ E, and from the marlstone vineyard (ND MAR) at 37° 39′ 18.5″ N, 12° 59′ 49.7″ E. SY from the limestone vineyard (SY LIM) was located at 37° 38′ 34.4″ N, 12° 58′ 26.4″ E, and from marlstone vineyard (SY MAR) at 37° 38′ 34.4″ N, 12° 58′ 26.4″ E  (Supporting Information, Figs S3 and S4). ND LIM soil was composed of 28% sand, 28.5% clay, and 43.3% silt. ND MAR was composed of 21% sand, 43.5% clay, and 35.5% silt. SY LIM was composed of 34.4% sand, 38.5% clay, and 27% silt. SY MAR was composed of 34.4% sand, 46% clay, and 20% silt (Supporting Information, Tables S5–S8).

The vineyards were distributed within a 10 km^2^ area, with a maximum distance of 2 km between individual sites. Both macroclimatic and microclimatic conditions could therefore be regarded as consistent across the vineyards during a given vintage. Meteorological data were recorded using a centralized weather station positioned at 37° 38′ 47.92” N and 12° 58′ 03.48″ E. Overall, the thermal conditions of the area corresponded to a subtropical temperate regime, and the precipitation pattern reflected a typical Mediterranean climate, characterized by peak rainfall occurring between October and January and a pronounced dry period lasting 5–6 months from May to September. All soil moisture regimes in the area were classified as Xeric, and soil temperature regimes were categorized as Thermic.^19^

All the experimental vineyards consisted of 30‐year‐old *Vitis vinifera* L. vines grafted onto 140 Ruggeri (*Vitis berlandieri* × *Vitis rupestris*) rootstock. Vines were planted at a spacing of 2.50 × 1.00 m on slopes of 5% to 15%, with south‐west to south‐east exposure, at an altitude of approximately 100 m a.s.l. Training followed the Guyot system, leaving one annual cane per plant tied to the fruiting wire parallel to the ground, along with a two‐bud spur for renewal. Suckering was performed before flowering, and no leaf‐removal practices were applied.

Irrigation was supplied via drip system with emitters spaced 0.6 m apart, delivering 4 L h^−1^, and maintaining stable soil moisture throughout ripening. Vineyards were managed under conventional agronomic practices, with harvest yields averaging 11–13 t ha^−1^ across both vintages.

### Sampling and sorting of grape berries by densimetric flotation

Grape berries of the CH, GR, ND and SY cultivars were collected in the 2021 and in 2022 vintages at technological maturity (white grapes at approximately 21 °Brix with a titratable acidity of 6–7 g L^−1^ of tartaric acid; red grapes at around 23 °Brix with a titratable acidity of 5–6 g L^−1^ tartaric acid). Approximately 2000 berries were randomly collected from each vineyard. Berries, with pedicels attached, were sampled from the middle and lower portions of clusters, both sun exposed and shaded, to capture the full range of ripeness in each vineyard.

Berries were floated following the method described by Bambina *et al*.^20^ To sort them by apparent density, salt solutions of varying concentrations were used. Six NaCl solutions were prepared, each increasing by 20 g L⁻¹, ranging from 90 to 190 g L⁻¹. Berries were first placed in the densest solution. Those that sank were considered to have a density equal to or slightly greater than that solution, while floating berries had lower densities. Sunken berries were immediately separated and counted, and floating berries were transferred to the next less dense solution. The procedure was repeated until all berries had sunk.

After density sorting, the berries were rinsed with water, inspected visually, and those with damaged skins were discarded. Berries in each density class were then weighed and counted. For further analysis, three subsamples of 20 berries from each density class were selected, as this number provides a representative sample while ensuring sufficient material to prepare skin and seed extracts for the analysis of phenolic composition.

The remaining berries were divided into three replicates, manually crushed, and centrifuged at 67 × *g* using a PK 131 centrifuge with a T516 swing‐out rotor (ALC International, Milano, Italy). The resulting supernatant was analyzed to determine key chemical and physical grape parameters within each density class. These parameters included reducing sugar content (g L^−1^), pH, and titratable acidity (g L^−1^). These measurements were conducted using a Winescan instrument (FOSS, Hillerød, Denmark), calibrated following the EEC 2676 standard procedure.^21^

### Preparation of extracts

Skins and seed extracts were prepared following the method outlined by Squadrito *et al*.^22^ Briefly, skins and seeds from 20 berries were separated from the pulp manually. Each component was placed in a separate plastic flask containing 20 mL of a pH 3.2 buffer solution, prepared with 5 g tartaric acid, 22.2 mL 1 mol L^−1^ NaOH, 2 g Na₂S₂O₅, 125 mL 95% ethanol, and deionized water to a final volume of 1 L.

Samples were maintained at room temperature for 4 h before homogenization at 83 × *g* for 1 min using an Ultraturrax T25 high‐speed homogenizer (IKA Labortechnik, Staufen, Germany). They were then subjected to an ultrasonic bath for 3 min and centrifuged at 67 × *g* using a PK 131 centrifuge with a T516 swing‐out rotor (ALC International). The supernatant was transferred to a 50 mL volumetric flask.

After each centrifugation, the pellet was resuspended in 5 mL of pH 3.2 buffer solution, for a total of three centrifugation cycles. All supernatants were combined and adjusted to a final volume of 50 mL using the buffer solution. The inclusion of Na₂S₂O₅ in the extraction buffer inhibited polyphenol oxidase activity, preventing oxidation of phenolic compounds and tissue browning. Analyses were performed in triplicate.

### Analysis of phenolic compounds

#### Skin and seeds flavonoids and total anthocyanins

Total flavonoids in skin and seeds, and total anthocyanins in skins, were measured at 280 nm and 540 nm, respectively, using UV–visible spectrophotometry, with a UV‐1800 spectrophotometer (Shimadzu Scientific Instruments Inc., Columbia, Maryland, USA). Skin and seed samples were thawed at room temperature for 30 min and homogenized for 1 min in tartaric buffer using an Ultra‐Turrax. Homogenates were subjected to ultrasound treatment for 3 min at room temperature and centrifuged at 67 × *g* for 15 min. The supernatant was collected and the pellet was resuspended in 5 mL of tartaric buffer, vortexed, and centrifuged again. This extraction was repeated three times and the combined supernatants were adjusted to a final volume of 50 mL with tartaric buffer.

Total flavonoid and anthocyanin content was determined after diluting extracts (the dilution factor was 50 for seeds extracts and 25 for skin extracts) in hydrochloric ethanol (ethanol:water:hydrochloric acid 37%, 70:30:1 v:v:v) according to the method proposed by Bambina *et al*.^20^ Results were expressed as mg kg^−1^ and analyses were performed in triplicate.

#### Analysis of hydroxycinnamoyl tartaric acids and flavonols

Grape skin extracts were acidified by adding 0.5 mL of 1 mol L^−1^ phosphoric acid (H₃PO₄) to 4.5 mL of extract to determine hydroxycinnamoyl tartaric acids (HCTAs) and flavonols. The acidified solutions were filtered through a 0.45 μm nylon membrane and transferred to 1.5 mL high‐performance liquid chromatography (HPLC) vials. Chromatographic analyses were performed using an HPLC system (Agilent 1200 Series, Milan, Italy) equipped with a diode array detector (Hewlett‐Packard 1100 DAD). Separation was carried out on a C18 reversed‐phase column (Econosphere C18, 5 μm, 250 × 4.6 mm i.d.), (Dr. Maisch, Ammerbuch‐Entringen, Germany). An injection volume of 20 μL was used following Squadrito *et al*.²²

To analyze HCTAs and flavonols, the mobile phase consisted of 10⁻³ M H₃PO₄ (solvent A) and CH₃OH (solvent B), varied during the analysis as follows: 5% B for 5 min; a linear gradient from 5% to 10% B in 5 min, 10% to 30% B in 10 min, 30% to 60% B in 10 min, and 60% to 100% B in 10 min; followed by a linear gradient from 100% to 5% B in 5 min. The flow rate was 0.48 mL min^−1^, and the column temperature was 40 °C. Hydroxycinnamoyl tartaric acids and flavonols were identified by comparing the retention times and absorption spectra of the pure compounds and laboratory‐isolated standards.^23^ Quantification was performed using calibration curves constructed with commercially available chlorogenic acid (for HCTAs) and quercetin (for flavonols) (Sigma‐Aldrich, St Louis, Missouri, USA; 95% purity). Results were expressed as mg kg⁻¹ of berries and analyses were performed in triplicate.

#### Technological variability index and the phenolic variability index

The technological variability index (TVI) and phenolic variability index (PVI), which measure grape heterogeneity, are constructed as aggregated, standardized indices that express absolute rather than relative variability. To combine this variability accurately, data were standardized so that each metabolite or physical characteristic contributed equally to overall variation. Absolute standardization was chosen over a relative approach, allowing comparisons across datasets regardless of units or scales, and facilitating the creation of variability databases. Metabolite standardization was performed using the coefficient of variation (CV):

$$\mathrm{CV}=\frac{\sigma }{\mu }$$

where σ is the sample standard deviation of the compound/physical–chemical parameter and μ is the sample mean of the compound/physical–chemical parameter.

The TVI can be defined as follows:

$$\mathrm{TVI}=CV_{\text{Reducing sugars}}+CV_{\mathrm{pH}}+CV_{\text{Titratable acidity}}$$

The PVI can be defined as follows:

$$\mathrm{PVI}=\begin{array}{l} CV_{\text{Caftaric acid}}+CV_{\text{coutaric acid}}+CV_{\text{Feftaric acid}}\\ +\;CV_{\text{Quercetin}-3-\text{glucoside}}+CV_{\text{skin}\;\mathrm{NAF}}+CV_{\text{seeds}\;\mathrm{NAF}}\\ +\;CV_{\text{anthocyanins}} \end{array}$$

Means and standard deviation were determined from all subsamples collected from each density class of berries separated by flotation using six NaCl solutions ranging from 90 to 190 g L^−1^, prepared at 20 g L^−1^ intervals.

## STATISTICAL ANALYSIS

A factorial analysis of variance (ANOVA) was applied to assess each factor's individual impact. The analysis was carried out using RStudio (Boston, Massachussets, USA) version 4.0.3. The main factors were grape variety, soil type, and vintage, with the interaction between the grape variety and soil type included. Differences with *P* values of less than 5% (*P* < 0.05) were considered statistically significant. When significant differences were detected, a *post hoc* Tukey's honestly significant difference (HSD) test was conducted. Results are expressed as means with corresponding standard errors. To highlight the soil effect among white and red cultivars grown on different calcareous soils, supervised partial least squares discriminant analyses (PLS‐DA) were performed for each group, separately, using the MetaboAnalyst web‐based tool suite.^24^

## RESULTS AND DISCUSSION

### Density classes distribution

**Figure 1:**
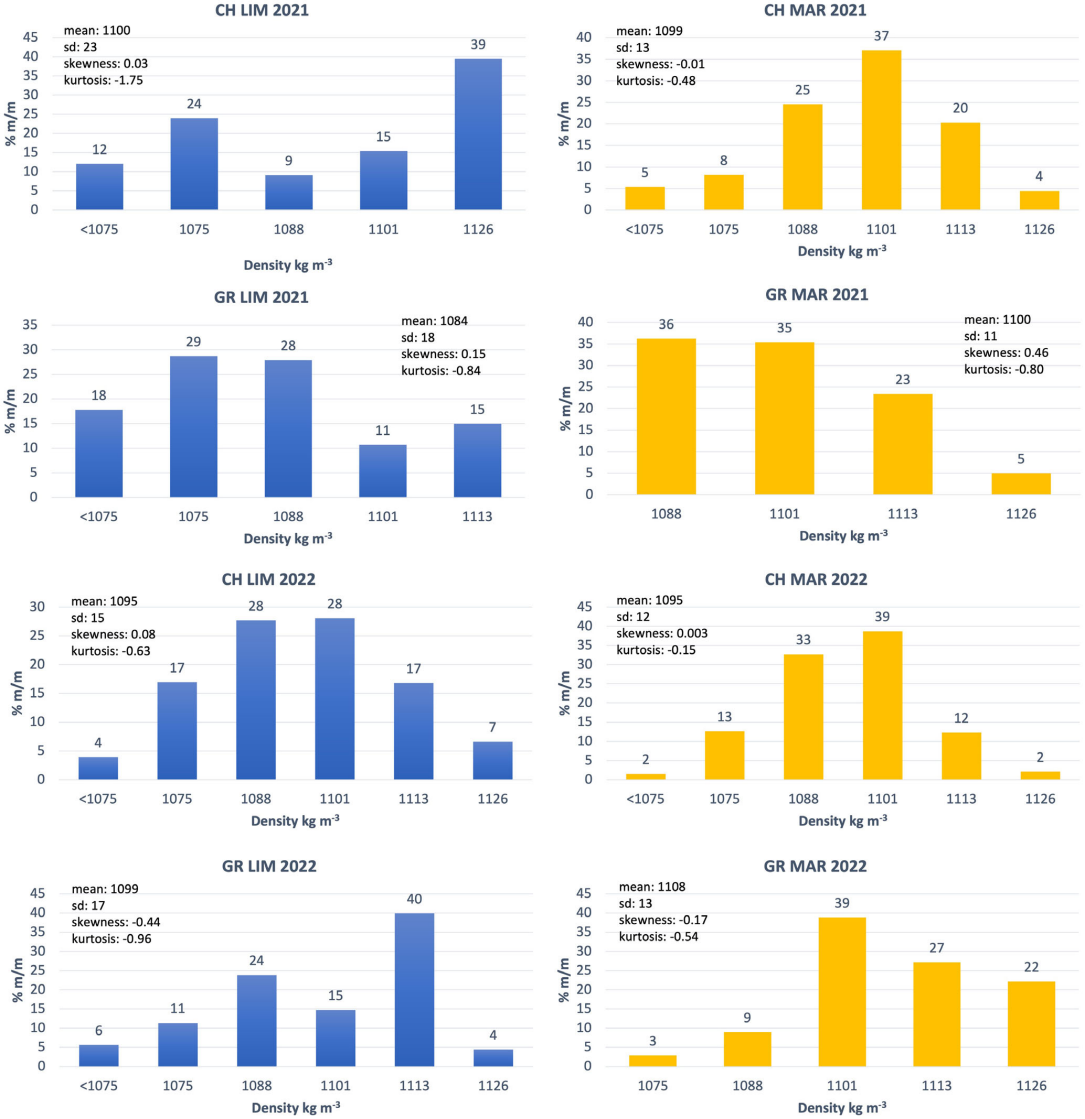
Density classes distributions of ‘Chardonnay’ and ‘Grillo’ grapes grown on different calcareous soils in the 2021 and 2022 vintages. CH LIM, ‘Chardonnay’ on limestone; CH MAR, ‘Chardonnay’ on marlstone; GR LIM, ‘Grillo’ on limestone; GR MAR, ‘Grillo’ on marlstone.

**Figure 2:**
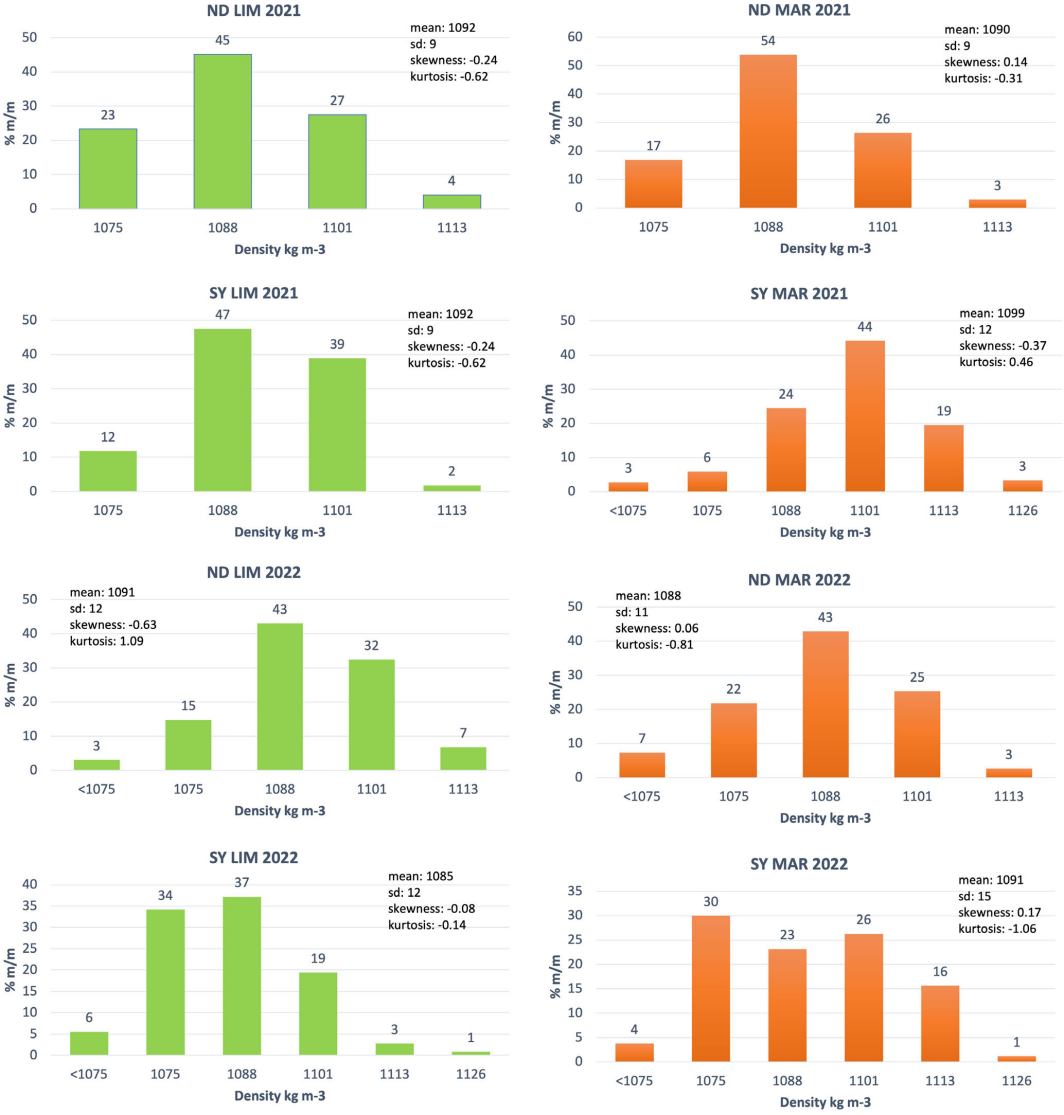
Density classes distributions of ‘Nero d’Avola’ (ND) and ‘Syrah’ (SY) grapes grown on different calcareous soils in the 2021 and 2022 vintages. ND LIM, ‘Nero d’Avola’ on limestone; ND MAR, ‘Nero d’Avola’ on marlstone; SY LIM, ‘Syrah’ on limestone; SY MAR, ‘Syrah’ on marlstone.

Flotation in salt solutions of varying concentrations revealed up to six density classes (<1075 to 1126 kg m⁻³), as shown in Fig. 1 for white grape cultivars and Fig. 2 for red cultivars, with statistical parameters describing the distribution of density classes for cultivars grown on different calcareous soils in the 2021 and 2022 vintages. Six density classes were adopted following the flotation study by Bambina *et al*.^20^

The distribution of grapes across density classes reflects ripeness heterogeneity at harvest, as berry density is correlated with reducing sugar content and therefore maturation stage. Each density class can thus be regarded as a distinct stage of berry maturation. The overall distribution was bell shaped.

The bell‐shaped distribution can be characterized by skewness, which indicates the maturation rate (positive skewness suggests delayed maturation, and negative skewness reflects early maturation) and kurtosis, which reflects the uniformity of maturation. Low kurtosis values indicate flatter distributions, with grapes distributed across multiple density classes, reflecting greater heterogeneity in ripeness. Conversely, higher kurtosis values correspond to sharper distributions, where dominant berry classes have similar ripening levels, indicating higher uniformity in grape maturation.

Analysis of berry density distributions (Table 1) revealed significant differences in ripening behavior and heterogeneity at harvest across cultivars, soils, and years. Cultivar strongly affected mean berry density, with GR and CH showing the highest values (1097 kg m^−3^), followed by SY; ND had significantly lower densities (1089.9 kg m^−3^), suggesting genotype‐specific ripening patterns. Skewness and kurtosis were not significantly influenced by cultivar, although a slight negative skewness in ND and SY indicated a higher proportion of less ripe berries. Vintage did not affect any distribution parameter significantly, suggesting stability between 2021 and 2022. Soil type significantly influenced mean, standard deviation, and median. Grapes grown on MAR soils reached higher mean densities and exhibited more homogeneous ripening (higher kurtosis), whereas LIM soils showed greater variability and more immature berries.

**Table 1:** Statistical parameters describing the distribution of the density classes as a function of variety, soil type, and vintage

Factor	Mean	Standard deviation	Median	Skewness	Kurtosis
Cultivar (*n* = 4)					
CH	1.097.31 ab	15.86 a	1100.6	0.02509589	−0.7505082
GR	1.097.78 a	14.46 a	1100.6	0.00166542	−0.7844814
ND	1.089.89 b	10.01 b	1087.9	−0.1981804	−0.2179848
SY	1.091.54 ab	11.84 ab	1091.075	−0.1346895	−0.3531417
Sign.	**	***	ns	ns	ns
Year (*n* = 8)					
2021	1094.28632	12.8289342	1094.25	−0.0245085	−0.6474532
2022	1093.97338	13.2549825	1095.8375	−0.1285458	−0.4056049
Sign.	ns	ns	ns	ns	ns
Soil (*n* = 8)					
LIM	1092.15094	14.2179751	1092.6625	−0.1706201	−0.5580062
MAR	1096.10876	11.8659415	1097.425	0.01756578	−0.4950519
Sign.	*	**	**	*	ns
Cultivar:soil (*n* = 2)					
CH:LIM	1.097.66 ab	19.27 a	1.100.60 ab	0.05	−1.19 b
GR:LIM	1.091.43 b	17.02 ab	1.094.25 bc	−0.14	−0.90 ab
ND:LIM	1.091.22 b	10.33 c	1.087.90 c	−0.44	0.23 a
SY:LIM	1.088.30 b	10.26 c	1.087.90 c	−0.16	−0.38 ab
CH:MAR	1.096.96 ab	12.45 bc	1.100.60 ab	0.00	−0.31 ab
GR:MAR	1.104.14 a	11.89 c	1.106.95 a	0.14	−0.67 ab
ND:MAR	1.088.56 b	9.70 c	1.087.90 c	0.04	−0.67 ab
SY:MAR	1.094.77 ab	13.42 bc	1.094.25 bc	−0.11	−0.33 ab
Sign.	**	***	*	ns	*

Note: Data are reported as means ± standard error of the mean. Sign. =ANOVA significance. Statistical significance is shown for each factor and for their interactions (ns, not significant; ·, *P* < 0.10; *, *P* < 0.05; **, *P* < 0.01; ***, *P* < 0.001). Different letters denote statistically significant differences based on post‐hoc tests (*P* < 0.05). CH, ‘Chardonnay’; GR, ‘Grillo’; ND, ‘Nero d’Avola’; SY, ‘Syrah’; LIM, limestone; MAR, marlstone.

Uneven ripening at harvest has substantial implications for wine composition and quality.^25^, ^26^ Tannin concentrations generally decrease during ripening, whereas underripe berries typically show higher acidity and tannin content. Grapes that meet technological maturity criteria (sugars, pH, acidity) may still contain a large proportion of underripe berries, rich in phenolics compounds and poor in grape‐derived glycosylated volatile compounds, crucial for wine quality.^14^ In red wines, phenolic composition, linked to phenolic maturity, strongly affects sensory attributes like color, structure, and astringency.^12^ These underripe grapes impart an astringent or ‘unripe’ character to the final wines, evoking the perception of an unripe fruit on the palate.^10^

Torchio *et al*.^4^ identified the 1073 kg m^−3^ density class as responsible for green and bitter wine notes. This class was prevalent in ND from LIM soils in 2021 (23%) and MAR soils in 2022 (22%), and in SY from MAR soils in 2022 (30%). It also appeared frequently in white grapes from LIM soils across both years.

Cultivar‐soil interaction influenced berry density. ‘Grillo’ on MAR soils showed the highest densities and most uniform ripening, whereas SY and ND on LIM soils had lower densities and flatter distributions. ND:LIM showed positive kurtosis, indicating ripening uniformity despite low density, and CH:LIM had the lowest kurtosis, reflecting high ripening variability. These findings suggest that the influence of soil characteristics on grape ripening heterogeneity is cultivar dependent and should be considered carefully in vineyard zoning and varietal selection strategies. Phenotypic plasticity varies among cultivars but some studies suggest that autochthonous varieties like ‘Aglianico’ are more influenced by environmental conditions than international varieties such as ‘Cabernet Sauvignon’.^27^

### Physicochemical parameters of grapes

The main physicochemical parameters of grapes, including reducing sugars (g L^−1^), titratable acidity (g L^−1^) and pH, are presented in Table 2. Among the cultivars, GR exhibited the highest reducing sugar concentration, followed by CH, ND, and SY. These differences were not statistically significant but they suggest a genotypic influence on sugar accumulation potential and ripening dynamics.

**Table 2:** Wine physical chemical composition

Factor	Reducing sugars g L^−1^	Titratable acidity g L^−1^	pH
Cultivar (*n* = 4)			
CH	225 ± 8	6 ± 1	3.5 ± 0.1 a
GR	241 ± 13	7.5 ± 0.4	3.2 ± 0.1 ab
ND	213 ± 6	8 ± 1	3.30 ± 0.04 bc
SY	208 ± 10	5.9 ± 0.3	3.4 ± 0.1 ab
Sign.	.	.	*
Year (*n* = 8)			
2021	227 ± 7	6.9 ± 0.5	3.4 ± 0.1
2022	217 ± 9	6.9 ± 0.4	3.4 ± 0.1
Sign.	ns	ns	ns
Soil (*n* = 8)			
LIM	214 ± 6	6.5 ± 0.4	3.4 ± 0.1
MAR	230 ± 9	7.2 ± 0.5	3.4 ± 0.1
Sign.	.	ns	ns
Cultivar:soil (*n* = 2)			
CH:LIM	229 ± 7	6 ± 1	3.4 ± 0.1 ab
GR:LIM	219 ± 5	7.7 ± 0.2	3.100 ± 0.001 c
ND:LIM	212 ± 6	6.9 ± 0.3	3.36 ± 0.01 bc
SY:LIM	194 ± 5	5.7 ± 0.1	3.55 ± 0.02 ab
CH:MAR	221 ± 12	7 ± 1	3.67 ± 0.01 ab
GR:MAR	262 ± 5	7 ± 1	3.3 ± 0.1 a
ND:MAR	214 ± 9	8.6 ± 0.3	3.23 ± 0.02 c
SY:MAR	222 ± 10	6.0 ± 0.5	3.32 ± 0.02 c
Sign.	ns	ns	**

Note: Data are reported as means ± standard error of the mean. Sign. =ANOVA significance. Statistical significance is shown for each factor and for their interactions (ns, not significant; ·, *P* < 0.10; *, *P* < 0.05; **, *P* < 0.01; ***, *P* < 0.001). Different letters denote statistically significant differences based on post‐hoc tests (*P* < 0.05). CH, ‘Chardonnay’; GR, ‘Grillo’; ND, ‘Nero d’Avola’; SY, ‘Syrah’; LIM, limestone; MAR, marlstone.

The titratable acidity ranged from 5.9 to 8.0 g L^−1^ among cultivars, with ND showing the strongest acidity. Cultivar had a statistically significant effect on pH. Chardonnay exhibited the highest pH, whereas GR and ND had lower values. Acidity and pH strongly influence wine quality by modulating microbial activity, stabilizing color in red wines, and affecting susceptibility to oxidation. They also play a key role in the evolution of volatile organic compounds, as esters responsible for fruity aromas hydrolyze faster under low pH conditions, whereas glycosidically bound aroma precursors are released through acid‐catalyzed hydrolysis. Acidity and pH also affect wine taste and mouthfeel significantly.^28^

Vintage had no significant influence on reducing sugars, acidity, or pH, indicating stability of compositional traits across the 2021 and 2022 seasons. Soil type, however, had a marginal effect on reducing sugars (*P* < 0.1), with grapes grown in MAR soils exhibiting higher sugar concentrations than those in LIM soils. No significant differences were observed for titratable acidity or pH between soil types.

A significant interaction effect was observed for pH, with the lowest values recorded in GR LIM and the highest in CH MAR. Although reducing sugars and acidity were not affected significantly by the interaction, GR grown in MAR soils gave the highest sugar content, suggesting a favorable cultivar–soil interaction for sugar accumulation.

Suter *et al*.^29^ showed that sugar accumulation in grape berries is strongly influenced by genetic background and genotype‐by‐environment interactions, with berry weight and veraison timing playing key roles alongside climatic factors such as temperature, radiation, and vine water status.

### Phenolic compounds

#### Total flavonoids, non‐anthocyanin flavonoids, and total anthocyanins

Table 3 reports the phenolic composition of the grape samples, including total flavonoids, non‐anthocyanin flavonoids (NAFs), the skin‐to‐seed NAF ratio, and anthocyanin content (in red cultivars only).

**Table 3:** Wine polyphenols content

Factor	FT mg kg^−1^	NAF mg kg^−1^	NAF skin/seeds	Anthocyanins mg kg^−1^
Cultivar (*n* = 4)				
CH	3.919 ± 246 ab	3.919 ± 246 a	0.25 ± 0.03 b	/
GR	2.177 ± 136 b	2.177 ± 136 b	0.27 ± 0.02 b	/
ND	5.365 ± 580 a	4.471 ± 455 a	0.58 ± 0.03 ab	623 ± 102 b
SY	5.051 ± 295 a	3.785 ± 253 a	0.7 ± 0.1 a	846 ± 23 a
Sign.	**	**	**	***
Year (*n* = 8)				
2021	3.875 ± 355	3.401 ± 269	0.5 ± 0.1	325 ± 131
S	4.381 ± 639	3.775 ± 448	0.4 ± 0.0	409 ± 158
Sign.	ns	ns	ns	ns
Soil (*n* = 8)				
LIM	4.197 ± 475	3.687 ± 353	0.4 ± 0.1	338 ± 135
MAR	4.059 ± 570	3.488 ± 395	0.5 ± 0.1	396 ± 156
Sign.	ns	ns	ns	ns
Cultivar:soil (*n* = 2)				
CH:LIM	4.295 ± 192 ab	4.295 ± 192	0.236 ± 0.004	/
GR:LIM	2.208 ± 215 b	2.208 ± 215	0.25 ± 0.02	/
ND:LIM	5.147 ± 215 ab	4.374 ± 128	0.572 ± 0.004	531 ± 60 b
SY:LIM	5.139 ± 397 ab	3.874 ± 337	0.6 ± 0.1	822 ± 8 a
CH:MAR	3.544 ± 54 ab	3.544 ± 54	0.27 ± 0.04	/
GR:MAR	2.146 ± 92 b	2.146 ± 92	0.28 ± 0.03	/
ND:MAR	5.583 ± 956 a	4.569 ± 772	0.6 ± 0.1	714 ± 139 a
SY:MAR	4.963 ± 310 ab	3.695 ± 266	0.7 ± 0.2	871 ± 30 a
Sign.	*	ns	ns	*

Note: Data are reported as means ± standard error of the mean. Sign. =ANOVA significance. Statistical significance is shown for each factor and for their interactions (ns, not significant; ·, *P* < 0.10; *, *P* < 0.05; **, *P* < 0.01; ***, *P* < 0.001). Different letters denote statistically significant differences based on post‐hoc tests (*P* < 0.05). CH, ‘Chardonnay’; GR, ‘Grillo’; ND, ‘Nero d’Avola’; SY, ‘Syrah’; LIM, limestone; MAR, marlstone. FT, total flavonoids; NAF, = non‐anthocyanic flavonoids.

As expected, red grape cultivars contained significantly higher levels of total flavonoids, NAFs, and anthocyanins than the white cultivars (CH and GR), in which anthocyanins were absent. The ratio of non‐anthocyanin flavonoids between skins and seeds also varied by cultivar, with Syrah showing the highest values (0.7 ± 0.1), indicating a greater contribution of skin tissues to total NAF, compared with ND (0.58 ± 0.003).

No significant effects of vintage or soil were detected when considered independently, although total flavonoid and anthocyanin content tended to be higher in the 2022 vintage and in grapes grown on MAR soils (396 mg kg⁻¹). Significant differences, however, arose from the interaction between cultivar and soil. Red cultivars on MAR soils accumulated more total flavonoids and anthocyanins than those on LIM soils, whereas white cultivars showed no soil‐related differences in flavonoid composition.

These results agree with Pollon *et al*.^1^ who observed that MAR soils induced a higher concentration of phenolic compounds in the grapes, such as anthocyanins and non‐anthocyanic flavonoids, compared with LIM soils. Ferrandino *et al*.^30^ further showed that finer textured soils, such as those rich in clay and silt, enhance anthocyanin and non‐anthocyanin flavonoid accumulation in grape berries. This effect is attributed to increased abscisic acid (ABA) synthesis, triggered by early drought signals in fine‐textured soils. Such soils impose moderate water stress initially, stimulating ABA production without causing severe stress. Abscisic acid subsequently promotes sugar and anthocyanin accumulation by modulating berry metabolism.^30^

Nevertheless, Bambina *et al*.^20^ found no significant correlation between soil texture components (sand, silt, and clay) and the anthocyanin content in ND wines, with the sole exception of cyanidin and silt, which showed a strong inverse relationship (Pearson's r = −0.98).

#### Hydroxycinnamoyl tartaric acid and flavonols

Hydroxycinnamoyl tartaric acids, including caftaric acid, coutaric acid, and feftaric acid, and flavonols including quercetin‐3‐glucuronide, quercetin‐3‐glucoside, and quercetin aglycon were detected in all the cultivars (Table 4).

**Table 4:** Hydrocinnamoyltartaric acids (HCTAs) and flavonols

Factor	Caftaric acid	Coutaric acid	Feftaric acid	Myricetin 3‐glucoside	Kaempferol 3‐glucoside	Quercetin 3‐glucuronide	Quercetin 3‐glucoside
Cultivar (*n* = 4)							
CH	370 ± 87 ab	231 ± 76	18 ± 3	0 ± 0 b	9 ± 1 b	27 ± 15	50 ± 10 b
GR	215 ± 58 b	81 ± 21	18 ± 5	0 ± 0 b	8 ± 3 b	14 ± 9	34 ± 9 b
ND	414 ± 125 a	52 ± 16	77 ± 41	62 ± 17 a	9 ± 4 b	32 ± 9	30 ± 11 b
SY	275 ± 34 ab	151 ± 54	178 ± 92	83 ± 23 a	61 ± 16 a	56 ± 8	122 ± 28 a
Sign.	*	ns	ns	**	**	ns	**
Year (*n* = 8)							
2021	389 ± 67	123 ± 47	127 ± 50	43 ± 20	29 ± 13	26 ± 11	77 ± 21
2022	248 ± 42	135 ± 33	19 ± 4	29 ± 12	15 ± 6	39 ± 6	40 ± 10
Sign.	**	ns	.	ns	.	ns	*
Soil (*n* = 8)							
LIM	320 ± 53	158 ± 52	70 ± 45	35 ± 19	24 ± 13	27 ± 9	61 ± 21
MAR	317 ± 70	100 ± 21	76 ± 37	37 ± 15	20 ± 6	38 ± 9	57 ± 13
Sign.	ns	ns	ns	ns	ns	ns	ns
Cultivar:soil (*n* = 2)							
CH:LIM	481 ± 99 ab	342 ± 68	20 ± 4	0 ± 0	8.2 ± 0.4	23 ± 16	58 ± 16
GR:LIM	268 ± 84 bc	98 ± 31	21 ± 9	0 ± 0	4 ± 1	9 ± 6	30 ± 13
ND:LIM	218 ± 32 bc	36 ± 3	34 ± 17	33 ± 3	3.8 ± 0.2	17 ± 1	13 ± 2
SY:LIM	311 ± 41 bc	156 ± 74	203 ± 127	108 ± 30	78 ± 20	59 ± 4	142 ± 31
CH:MAR	259 ± 21 bc	120 ± 16	17 ± 0	0 ± 0	10.5 ± 0.4	31 ± 21	41 ± 1
GR:MAR	161 ± 18 c	65 ± 8	15 ± 2	0 ± 0	12 ± 2	19 ± 13	38 ± 9
ND:MAR	610 ± 85 a	69 ± 22	120 ± 54	91 ± 5	14 ± 4	48 ± 3	47 ± 6
SY:MAR	239 ± 19 bc	147 ± 56	153 ± 93	58 ± 9	44 ± 10	54 ± 13	101 ± 31
Sign.	**	ns	Ns	.	ns	ns	.

Note: Data are reported as means ± standard error of the mean. Sign. =ANOVA significance. Statistical significance is shown for each factor and for their interactions (ns, not significant; ·, *P*  < 0.10; *, *P* < 0.05; **, *P* < 0.01; ***, *P* < 0.001). Different letters denote statistically significant differences based on post‐hoc tests (*P* < 0.05). CH, ‘Chardonnay’; GR, ‘Grillo’; ND, ‘Nero d’Avola’; SY, ‘Syrah’; LIM, limestone; MAR, marlstone.

Hydrocinnamoyltartaric acids and flavonols are expressed in mg kg^−1^.

Caftaric acid is important because it is involved in the browning of musts and wines, serving as the preferred substrate for grape tyrosinase, which converts it into reactive quinones.^31^ Hydroxycinnamoyl tartaric acids vary with grape variety and maturation level.^32^ These quinones are highly reactive toward nucleophilic attacks by sulfur dioxide and sulfur‐containing aromatic compounds, leading to musts and wines with reduced varietal aroma and decreased effectiveness of sulfur dioxide as a protective agent.^32^

The most abundant flavonol was quercetin‐3‐glucoside in GR, CH and SY, whereas in ND it was myricetin‐3‐glucoside. Flavonols contribute to wine quality by acting as antioxidants and as co‐pigments for anthocyanins in red wines.^33^, ^34^ In white wines, early separation of grape skins from the must limits the extraction of these compounds.^35^ The higher concentrations of quercetin‐3‐glucoside observed in the 2021 vintage compared with 2022 can be attributed to the fact that the biosynthesis of this compound is strongly influenced by abiotic stresses, particularly solar radiation and drought conditions.^36^ The 2021 season was characterized by elevated temperatures and prolonged dry periods.

Significant differences among cultivars were observed for several compounds. ‘Nero d'Avola’ and CH had the highest concentrations of caftaric acid (414 ± 125 and 370 ± 87 mg kg⁻¹, respectively), with a statistically significant effect (*P* < 0.05). Although SY also exhibited a high mean value (275 ± 34 mg kg⁻¹), GR was significantly lower (215 ± 58 mg kg⁻¹). Myricetin‐3‐glucoside was absent in white cultivars, whereas red cultivars accumulated substantial amounts. ‘Syrah’ had the highest contents of myricetin‐3‐glucoside (83 ± 23 mg kg⁻¹) and kaempferol‐3‐glucoside (61 ± 16 mg kg⁻¹), followed by ND. Quercetin‐3‐glucoside also varied significantly among cultivars, with SY showing the highest value (122 ± 28 mg kg⁻¹), significantly greater than both white cultivars and ND (30 ± 11 mg kg⁻¹).

Vintage significantly influenced some compounds. Caftaric acid was higher in 2021 (389 ± 67 mg kg⁻¹) than in 2022 (248 ± 42 mg kg⁻¹; *P* < 0.01), whereas quercetin‐3‐glucoside was also higher in 2021 (77 ± 21 mg kg⁻¹) compared with 2022 (40 ± 10 mg kg⁻¹; *P* < 0.05). No significant differences were observed between LIM and MAR soils for any compound. However, significant cultivar–soil interactions were detected for caftaric acid (*P* < 0.01), myricetin‐3‐glucoside, and quercetin‐3‐glucoside.

The highest concentration of caftaric acid was observed in ND MAR (610 ± 85 mg kg⁻¹), followed by CH LIM (481 ± 99 mg kg⁻¹). Myricetin‐3‐glucoside peaked in SY LIM (108 ± 30 mg kg⁻¹) but declined markedly in the same cultivar on MAR soil (58 ± 9 mg kg⁻¹). SY LIM also exhibited the highest quercetin‐3‐glucoside (142 ± 31 mg kg⁻¹), highlighting its potential for flavonol accumulation under specific soil conditions.

### Technological variability index (TVI) and phenolic variability index (PVI)

The coefficients of variation reported in Supporting Information, Table S17 were calculated for key oenological parameters and major phenolic compounds, including HCTAs and flavonols, throughout the ripening process (Supporting Information, Tables S9–S16). Reducing sugars and pH increased with apparent density, whereas total acidity, tartaric acid and malic acid decreased with maturation (Supporting Information, Table S13). Skin total flavonoids and anthocyanins tended to increase during ripening and slightly decrease in the final density class, whereas seed flavonoids decreased with increasing ripeness (Supporting Information, Table S14).

Most flavonoids, except anthocyanins, are synthesized from early berry development until veraison,^37^ after which their concentrations generally remain stable or decrease slightly due to oxidative and thermal degradation.^38^ The increase in total skin flavonoids observed here may therefore reflect greater extractability linked to histochemical changes during ripening,^39^ whereas in seeds lignification reduces extractability in riper grapes.^40^

The coefficient of variation (CV) for the studied variables was calculated as the ratio between the standard deviation and the weighted mean obtained from the different density classes of each sample. The coefficient of variation is a statistical measure that represents the degree of relative variability of a dataset in relation to its mean. It is commonly used to compare the dispersion of variables with different scales or units of measurement, making it a valuable indicator in comparative analyses.

The TVI and phenolic variability index PVI indices were calculated as the sum of the coefficients of variation of basic parameters and phenolic compounds, respectively (Supporting Information, Table S17). The TVI and PVI are reported in Table 5. The TVI provides insights into within‐vineyard variability at harvest based on basic technological parameters (reducing sugars, pH, titratable acidity), whereas the PVI reflects variability in phenolic compounds, including caftaric, coutaric, and feftaric acids, quercetin‐3‐glucoside, skin NAF, seed NAF, and total anthocyanins).

The development of the TVI and the PVI provided an integrated, quantitative assessment of grape ripening heterogeneity, effectively capturing differences in technological and phenolic maturity among vineyard parcels. These indices allow evaluation of grape heterogeneity at harvest and the stability of a variety's traits under different conditions, such as soil type. Low TVI or PVI values indicate greater homogeneity in grape ripening, which is beneficial for winemaking because uniform grape composition generally improves wine quality.^4^ For example, low variability in sugar content, acidity, and phenolic compounds at harvest reduces the likelihood of negative effects on the chemical composition and sensory profile of the resulting wine.^27^

The stability of these indices across vintages is equally important. If the TVI and PVI remain stable despite climatic differences between vintages, it demonstrates the plasticity of the variety. Plasticity refers to the ability of a grape variety to adapt to changing environmental conditions, such as variations in temperature, rainfall, or soil moisture.^9^ This adaptability is a valuable trait, especially in the context of climate change, where weather patterns are increasingly unpredictable.^12^ Furthermore, TVI and PVI values can also provide information about how specific grape varieties interact with different soil types.

In Table 5 significant differences in TVI were observed among the four grape cultivars (*P* < 0.05). ‘Syrah’ exhibited the highest TVI (0.25 ± 0.02), followed by CH (0.20 ± 0.1) and GR (0.21 ± 0.03), whereas ND had the lowest value (0.14 ± 0.01), indicating lower heterogeneity of ripening at harvest. The maximum TVI difference observed was 0.11, which is modest but still reflects measurable variation in technological maturity. Even relatively small deviations in grape ripening can affect must composition and influence harvest management.

**Table 5:** Technological variability index (TVI) and phenolic variability index (PVI)

Factor	TVI	PVI
Cultivar (*n* = 4)		
CH	0.2 ± 0.1 ab	4 ± 1
GR	0.21 ± 0.03 bc	3 ± 1
ND	0.14 ± 0.01 c	4 ± 1
SY	0.25 ± 0.02 a	3.7 ± 0.3
Sign	*	ns
Year (*n* = 8)		
2021	0.22 ± 0.03	4 ± 1
2022	0.19 ± 0.02	3.6 ± 0.3
Sign	ns	ns
Soil (*n* = 8)		
LIM	0.23 ± 0.03	4.1 ± 0.3
MAR	0.18 ± 0.02	3.4 ± 0.5
Sign	*	ns
Cultivar:Soil (*n* = 2)		
CH:LIM	0.3 ± 0.1 ab	5 ± 2
GR:LIM	0.25 ± 0.03 abc	3.9 ± 0.2
ND:LIM	0.13 ± 0.02 c	4 ± 1
SY:LIM	0.22 ± 0.05 ab	4 ± 1
CH:MAR	0.12 ± 0.01 bc	4 ± 2
GR:MAR	0.16 ± 0.03 abc	2 ± 1
ND:MAR	0.15 ± 0.04 c	4 ± 2
SY:MAR	0.281 ± 0.001 a	4 ± 1
Sign	**	ns

Note: Data are reported as means ± standard error of the mean. Sign. =ANOVA significance. Statistical significance is shown for each factor and for their interactions (ns, not significant; ·, *P* < 0.10; *, *P* < 0.05; **, *P* < 0.01; ***, *P* < 0.001). Different letters denote statistically significant differences based on post‐hoc tests (*P* < 0.05). CH, ‘Chardonnay’; GR, ‘Grillo’; ND, ‘Nero d’Avola’; SY, ‘Syrah’; LIM, limestone; MAR, marlstone.

Higher TVI and PVI values indicate less homogeneous grape ripening, which can result in uneven distributions of sugars, acids, phenolic, and aromatic compounds among berries. Such heterogeneity may, in turn, affect the balance between sugar and acidity, the degree of phenolic maturity, and the extractability of color and tannins during winemaking. Consequently, TVI provides not only a statistical measure of variability but also a practical indicator of potential differences in wine composition, structure, and sensory expression arising from vineyard heterogeneity. No statistically significant differences were found in PVI across cultivars, which ranged between 3 and 4.

Soil type significantly influenced TVI (*P* < 0.05), with grapes grown on LIM soils showing higher TVI (0.23 ± 0.03) than those on MAR soils (0.18 ± 0.02). This suggests a more pronounced heterogeneity of grape ripening in LIM soils, although PVI remained statistically unaffected. These findings agree with Bigard *et al*,^6^ and Rolle *et al*,^10^ who reported that terroir affects grape sugar variability by comparing berry technological parameters and observing differences in berry flotation among regions. No significant differences were found between the 2021 and 2022 vintages.

A significant cultivar–soil interaction was observed for TVI (*P* < 0.01). The highest TVI was recorded in SY MAR (0.281 ± 0.001), followed by CH LIM (0.30 ± 0.1), whereas ND maintained low TVI values regardless of soil type. PVI values showed no significant interaction and remained stable across all cultivar–soil combinations.

Although some studies suggest that international varieties are generally more resilient,^41^ the present findings indicate that autochthonous grape varieties exhibited greater adaptability to their native environments than international cultivars. This is reflected in the higher stability of technological and phenolic parameters across diverse pedoclimatic conditions. Phenotypic plasticity is a key trait for grapevine adaptation to climate change, requiring a multi‐trait approach to account for cultivar‐specific responses to environmental variability.^29^

Lower TVI and PVI values, indicating greater homogeneity of grapes in technological parameters and phenolic compounds, correspond to higher kurtosis and more positive skewness of the berry distribution. These parameters suggest that berry distributions are more homogeneous as ripening progresses. This trend is more pronounced in grapes grown on MAR soils than on LIM soils, although the values observed for LIM remain moderate and still permit the production of high‐quality wines.

Based on TVI and PVI scores, grape variability decreased progressively as berries advanced toward full ripening, indicating that harvesting too early, even when technological maturity is achieved, may lead to greater heterogeneity.^20^ This trend can be explained by the fact that, under favorable climatic conditions supporting effective photosynthetic activity, growth‐related genes are overexpressed in less mature berries, enabling them to reach the ripening stage of more advanced berries.^7^ Consequently, harvest achieves a more homogeneous grape maturity. In contrast, prolonged periods of high temperatures and drought can inhibit photosynthetic activity, thereby reducing the homogeneity of grape ripening.

Similarly, earlier ripening, which shifts the ripening period to a time of the season when climatic conditions are less favorable for high‐quality wine production, can hinder the uniform development of berries and result in increased heterogeneity at harvest.^41^ Variability in sugar accumulation, acidity, and phenolic composition within the same vineyard parcel, can lead to differences in the resulting wine quality and style outcomes.^9^, ^27^ Quantifying grape variability through composite indices like TVI and PVI provides an important tool for optimizing harvest timing and tailoring winemaking practices. Monitoring the stability or variability of these indices over time is therefore essential to better understand the interaction between grapevine genotype, soil, and environmental conditions.

The TVI and PVI indices are strategic tools that not only support informed decision‐making but also provide a basis for interventions aimed at improving ripening uniformity over time. Integrating these indices into predictive models and databases that incorporate climatic conditions would further enhance their utility, allowing growers to anticipate the effects of environmental variability on grape composition. This approach could ultimately improve harvest timing, optimize winemaking practices, and support the development of decision‐support systems for viticulture under changing climate scenarios.

### The relationship between soil and grapes

**Figure 3:**
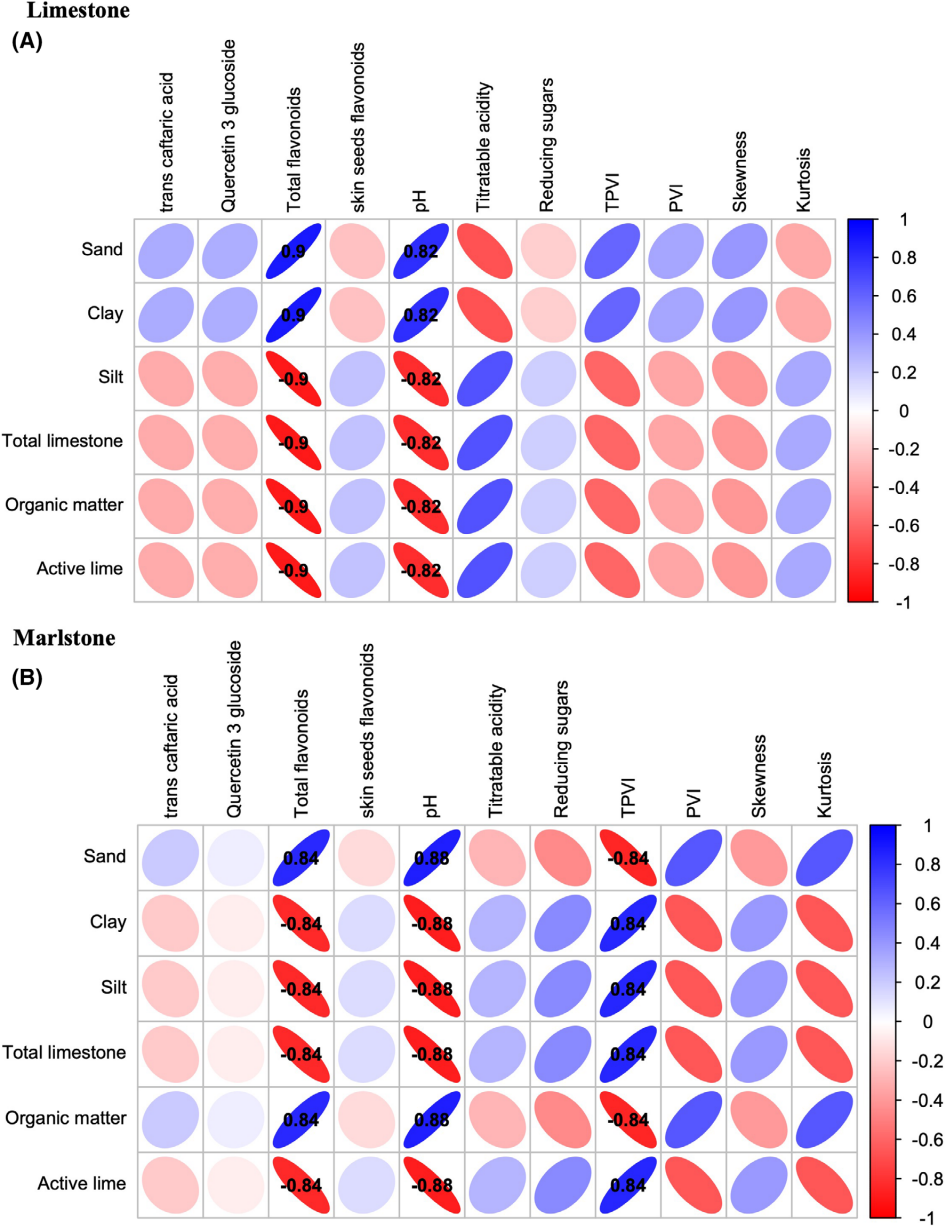
Correlation heatmap between soil chemical–physical parameters and the compositional characteristics of ‘Chardonnay’, ‘Grillo’, ‘Nero d’Avola’ and ‘Syrah’ grapes grown on limestone (A) and marlstone (B) soils. Correlations are represented using a color scale ranging from red (negative correlation) to blue (positive correlation), with color intensity proportional to the strength of the correlation. Only significant correlations with *Pearson's* r values > |0.80| and *P* < 0.05, are displayed in heatmaps.

Soil is a key environmental factor influencing grapevine development and determining grape and wine quality.^42^ Fig 3 presents a correlation heatmap showing soil chemical–physical parameters and some compositional characteristics of CH, GR, ND, and SY grapes grown on limestone (A) and marlstone (B) soils. The correlation heatmap reports correlations between the soils’ chemical and physical parameters (Supporting Information, Tables S1–S8) and the most important grape characteristics. Only significant correlations (Pearson's r > |0.80| and *P* < 0.05) are indicated numerically in the figures. Certain similarities exist between soil parameters and grape compositional variables and notable differences can be seen in their respective correlations.

In LIM soils, sand and clay are positively correlated with total flavonoids and pH, whereas silt, total limestone, organic matter, and active lime are negatively correlated with these variables. A similar pattern is observed in marlstone soils, although clay and organic matter exhibit opposite trends. However, Bambina *et al*.^20^ did not find significant correlations between soil texture parameters (clay, silt, and sand) and the phenolic composition of ND grapes.

In Ferrandino *et al*.'s^30^ work on the ‘Nebbiolo’ cultivar, higher concentrations of phenolic compounds were attributed to increased ABA, stimulated by finer soil particles such as clay and silt compared with coarser sand particles. Several soil parameters in marlstone soils also showed significant correlations with the TVI value: sand and organic matter were negatively correlated, whereas clay, silt, total limestone, and active lime were positively correlated.

Several studies have shown that soil physical and chemical properties critically influence vine growth, grape ripening, and the compositional attributes of the resulting wine.^20^, ^43^ The proportions of sand, silt, and clay in the soil affect its ability to retain water, exchange cations, allow root penetration, and regulate root temperature. Fine‐textured soils tend to induce greater initial water stress, whereas at later phenological stages, coarse‐textured soils impose more severe stress due to their lower water‐holding capacity. This dynamic soil–plant interaction highlights the importance of soil texture in modulating stress responses and secondary metabolite accumulation in grapevines.^44^

The differential progression of soil water potential across varying soil textures suggests that vines rooted in fine‐textured substrates can perceive drought signals even under moderate soil moisture conditions. This early perception promotes increased accumulation of ABA. This phytohormone has been shown to enhance the accumulation of sugars and phenolic compounds in grape berries by modulating key metabolic pathways.^44^

### Multivariate statistical analysis

To assess the effect of soil type (LIM and MAR) on grape composition, a supervised partial least squares discriminant analysis (PLS‐DA) was performed separately for white and red grape cultivars, allowing the visualization of spatial separation between samples based on their overall chemical composition.

**Figure 4:**
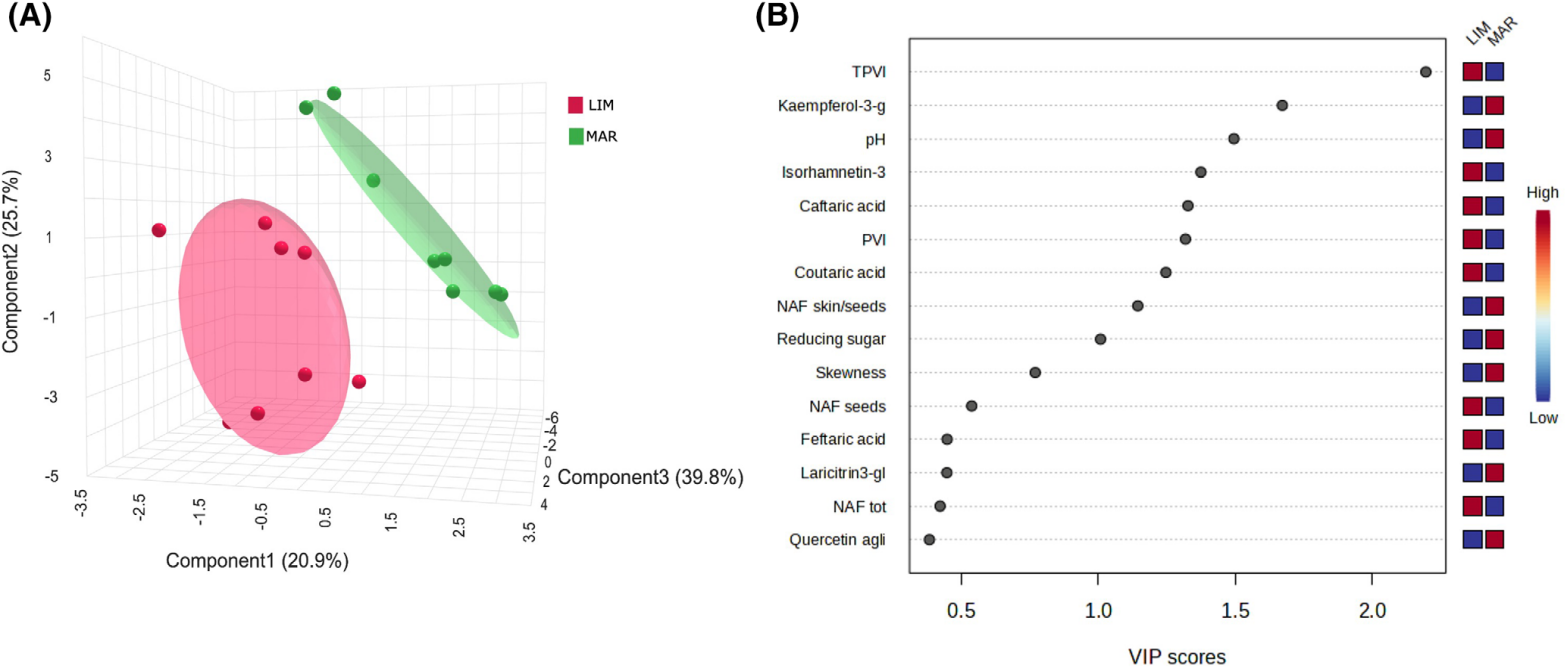
(A) Three‐dimensional partial least squares discriminant analysis (PLS‐DA) score plot showing the separation of grape samples based on soil type: LIM (limestone) in purple and MAR (marlstone) in green. The analysis includes the white grape cultivars (‘Chardonnay’ and ‘Grillo’). (B) Variable importance in projection (VIP) scores highlighting the most discriminant variables contributing to the separation between soil types.

The PLS‐DA score plot (Fig. 4(A)) clearly distinguishes the white grape samples (CH and GR) according to soil type, with distinct clustering observed for LIM and MAR groups. This separation indicates that soil type exerted a strong influence on the compositional traits of the grapes. The PLS‐DA analysis reduced the number of original variables to three components that explained 86.4% of the total variance of the dataset. The corresponding VIP plot (Fig. 4(B)) identified the most influential variables contributing to group discrimination. The colored boxes on the right of the plot indicate the relative concentrations of the metabolites in soil type.

Even for the red cultivars, PLS‐DA analysis reduced the number of original variables to three components, explaining 97.7% of the variance in the dataset. The scores plot shown in Fig. 4 (A) shows two distinct clusters indicating the effect of soil type on the composition of ND and SY samples.

According to the VIP scores in Fig. 4(B) (white grape varieties), TVI and PVI were the key variables driving the separation between soil types. These indices were higher in grapes grown on LIM soils, whereas MAR soils were associated with higher skewness, pH, and phenolic compounds such as quercetin.

**Figure 5:**
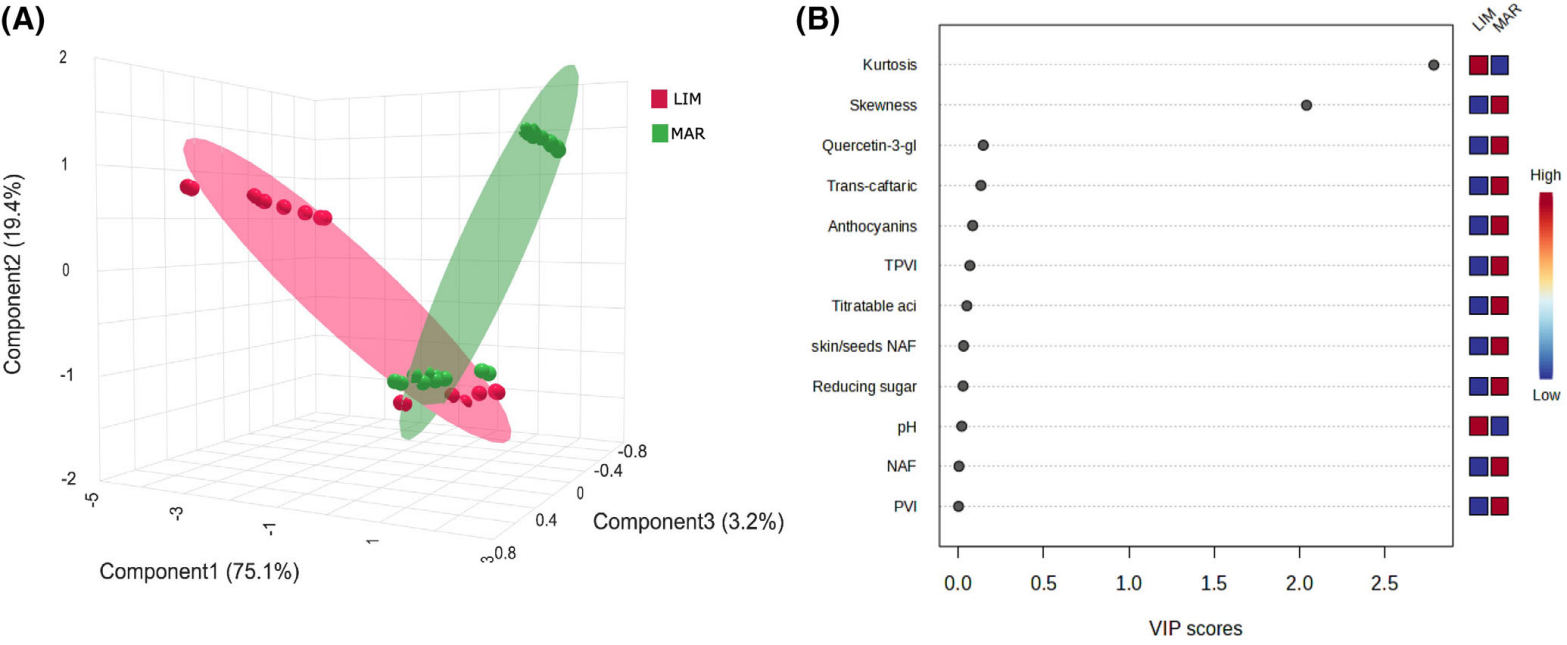
(A) Three‐dimensional partial least squares discriminant analysis (PLS‐DA) score plot showing the separation of grape samples based on soil type: LIM (limestone) in purple and MAR (marlstone) in green. The analysis includes the red grape cultivars (‘Nero d’Avola’ and ‘Syrah’). (B) Variable importance in projection (VIP) scores highlighting the most discriminant variables contributing to the separation between soil types.

In contrast, the VIP scores in Fig. 5(B) (red grape varieties) indicate a different soil‐dependent pattern. Grapes from LIM soils exhibited higher pH and kurtosis values, whereas MAR soils were associated with greater skewness, higher TVI and PVI indices, and elevated concentrations of phenolic compounds, including anthocyanins, and quercetin.

These results underscore the strong interaction between genotype and soil type, highlighting how soil chemical–physical parameters can differentially influence ripening dynamics and phenolic composition in white and red grape cultivars, conceivably due to its influence on water availability, nutrient dynamics, and vine vigor throughout the growing season.^20^

To prevent data overfitting with PLS‐DA and to ensure the reliability of the analysis, permutation test and cross validation were applied (Supporting Information, Figs S5 and S6). Cross‐validation (Figs S5 and S6A) confirmed the model's significance and predictability for both white cultivars (accuracy = 1; R² = 1; Q² = 1) and red cultivars (accuracy = 1; R² = 0.9; Q² = 0.9). The *P* values from the permutation tests (*n* = 1000) were 0.001 (Figs S5 and S6B)

## CONCLUSION

This study highlights the key role of soil type in determining the physicochemical and phenolic characteristics of white (CH and GR) and red (ND and SY) cultivars grown on calcareous soils (limestone and marlstone) in southwestern Sicily.

The technological parameter variability index (TVI) and the phenolic variability index (PVI) were proposed as summary tools to describe overall grape heterogeneity. The TVI is calculated as the sum of coefficients of variation across berry density classes for reducing sugars, pH, and titratable acidity, whereas the PVI aggregates coefficients of variation for caftaric acid, coutaric acid, feftaric acid, quercetin‐3‐glucoside, skin NAF, seed NAF, and anthocyanins. Both indices effectively quantified grape variability across environmental and varietal factors, providing valuable insights for vineyard management, winemaking, and quality prediction.

Grapes from marlstone soils exhibited lower TVI values and a more uniform berry maturation, as indicated by higher kurtosis and more positive skewness indices, suggesting that variability decreased throughout ripening as berries reached greater maturity. These findings highlight that harvesting fruit too early, even if technologically mature, may increase grape heterogeneity. Conversely, grapes grown on limestone soils were associated with greater technological variability and higher PVI values.

Sicilian varieties, both white and red, showed lower ripening heterogeneity than international cultivars across the different calcareous soils, indicating greater adaptability and a more uniform maturation process in response to their native pedoclimatic conditions. Multivariate statistical analyses further confirmed the dominant influence of soil in differentiating grape composition.

Overall, these findings demonstrate that the TVI and PVI indices are effective tools for supporting precision viticulture, optimizing harvest timing, and guiding winemaking strategies under increasing climatic variability. However, the study is limited by the relatively small number of vineyards and grape varieties sampled. Future research should therefore extend this approach to a broader range of cultivars and wine‐growing regions to better assess the role of soil type in grape ripening variability and its impact on the chemical and sensory properties of the resulting wine.

## AUTHOR CONTRIBUTIONS

Clara Vitaggio: conceptualization; methodology; investigation; formal analysis; data curation; writing – original draft. Matteo Pollon: investigation; formal analysis; data curation. Manuel Schnitter: investigation; formal analysis. Valentina Caraci: formal analysis. Luciano Cinquanta: conceptualization; methodology; data curation; writing – review and editing. Onofrio Corona: conceptualization; methodology; data curation; writing – review and editing.

## FUNDING INFORMATION

This study was funded by ‘Programma di Sviluppo Rurale Sicilia 2014–2020’, Misura16 – Sottomisura 16.1 Sostegno per la costituzione e la gestione dei gruppi operativi del PEI in materia di produttività e sostenibilità dell’agricoltura’ (Bando 2018), project title ‘Tecniche viticole ed enologiche innovative e sostenibili per la valorizzazione dei suoli calcarei (VEISCA)’, CUP (Codice Unico di Progetto) B93C22001440005.

## CONFLICT OF INTEREST

The authors declare no conflict of interest.

## Supporting information


**Table S1.** Chemical and physical parameters of the limestone soil (CH LIM) where ‘Chardonnay’ vines are cultivated.
**Table S2.** Chemical and physical parameters of the marlstone soil (CH MAR) where ‘Chardonnay’ vines are cultivated.
**Table S3.** Chemical and physical parameters of the limestone soil (GR LIM) where ‘Grillo’ vines are cultivated.
**Table S4.** Chemical and physical parameters of the marlstone soil (GR MAR) where ‘Grillo’ vines are cultivated.
**Table S5.** Chemical and physical parameters of the limestone soil (ND LIM) where ‘Nero d’Avola’ vines are cultivated.
**Table S6.** Chemical and physical parameters of the marlstone soil (ND MAR) where ‘Nero d’Avola’ vines are cultivated.
**Table S7.** Chemical and physical parameters of the limestone soil (SY LIM) where ‘Syrah’ vines are cultivated.
**Table S8.** Chemical and physical parameters of the marlstone soil (SY MAR) where ‘Syrah’ vines are cultivated.
**Table S9.** Technological parameters content during ripening of Chardonnay and Grillo cultivar grown on limestone and marlstone soils in 2021 and 2022 vintages.
**Table S10.** Skin and seeds total flavonoids content during ripening of ‘Chardonnay’ and ‘Grillo’ cultivar grown on limestone and marlstone soils in 2021 and 2022 vintages.
**Table S11.** Flavonols content during ripening of ‘Chardonnay’ and ‘Grillo’ cultivar grown on limestone and marlstone soils in 2021 and 2022 vintages.
**Table S12.** Hydrocycinnamoyltartaric acids (HCTAs) content during ripening of ‘Chardonnay’ and ‘Grillo’ cultivar grown on limestone and marlstone soils in 2021 and 2022 vintages.
**Table S13.** Technological parameters content during ripening of ‘Nero d’Avola’ and ‘Syrah’ cultivar grown on limestone and marlstone soils in 2021 and 2022 vintages.
**Table S14.** Skin and seeds total flavonoids and anthocyanins content during ripening of ‘Nero d’Avola’ and ‘Syrah’ cultivar grown on limestone and marlstone soils in 2021 and 2022 vintages.
**Table S15.** Flavonols content during ripening of ‘Nero d’Avola’ and ‘Syrah’ cultivar grown on limestone and marlstone soils in 2021 and 2022 vintages.
**Table S16.** Hydrocycinnamoyltartaric acids (HCTAs) content during ripening of ‘Nero d’Avola’ and ‘Syrah’ cultivar grown on limestone and marlstone soils in 2021 and 2022 vintages.
**Table S17.** Coefficient variation of some technological parameters and phenolic compounds.
**Fig. S1.** Satellite images of the two vineyard parcels used in the study for ‘Chardonnay’ cultivar: limestone soil (CH LIM) (A) and marlstone soil (CH MAR) (B), both located in southwestern Sicily. The coordinates of each vineyard are indicated.
**Fig. S2.** Satellite images of the two vineyard parcels used in the study for ‘Grillo’ cultivar: limestone soil (GR LIM) (A) and marlstone soil (GR MAR) (B), both located in southwestern Sicily. The coordinates of each vineyard are indicated.
**Fig. S3.** Satellite images of the two vineyard parcels used in the study for ‘Nero d’Avola’ cultivar: limestone soil (ND LIM) (A) and marlstone soil (ND MAR) (B), both located in southwestern Sicily. The coordinates of each vineyard are indicated.
**Fig. S4.** Satellite images of the two vineyard parcels used in the study for ‘Syrah’ cultivar: limestone soil (SY LIM) (A) and marlstone soil (SY MAR) (B), both located in southwestern Sicily. The coordinates of each vineyard are indicated.
**Fig. S5.** Permutation test (B) and cross validation (A) (n = 1000) for the PLS‐DA analysis carried out on white grapes (‘Chardonnay’ and ‘Grillo’) composition.
**Fig. S6.** Permutation test (B) and cross validation (A) (n = 1000) for the PLS‐DA analysis carried out on red grapes (‘Nero d’Avola’ and ‘Syrah’) composition.

## Data Availability

The data that supports the findings of this study are available in the supplementary material of this article.
