# Supplementary material for: Grape ripening heterogeneity in white and red cultivars ( Vitis vinifera L.) grown on different calcareous soils using technological and phenolic markers
Source: J Sci Food Agric. 2025 Oct 8;106(2):1079–93. doi: 10.1002/jsfa.70231 (PMC12701293; doi:10.1002/jsfa.70231)
Supplement: Supplementary file 1 — Table S1. Chemical and physical parameters of the limestone soil (CH LIM) where ‘Chardonnay’ vines are cultivated. Table S2. Chemical and physical parameters of the marlstone soil (CH MAR) where ‘Chardonnay’ vines are cultivated. Table S3. Chemical and physical parameters of the limestone soil (GR LIM) where ‘Grillo’ vines are cultivated. Table S4. Chemical and physical parameters of the marlstone soil (GR MAR) where ‘Grillo’ vines are cultivated. Table S5. Chemical and physical parameters of the limestone soil (ND LIM) where ‘Nero d’Avola’ vines are cultivated. Table S6. Chemical and physical parameters of the marlstone soil (ND MAR) where ‘Nero d’Avola’ vines are cultivated. Table S7. Chemical and physical parameters of the limestone soil (SY LIM) where ‘Syrah’ vines are cultivated. Table S8. Chemical and physical parameters of the marlstone soil (SY MAR) where ‘Syrah’ vines are cultivated. Table S9. Technological parameters content during ripening of Chardonnay and Grillo cultivar grown on limestone and marlstone soils in 2021 and 2022 vintages. Table S10. Skin and seeds total flavonoids content during ripening of ‘Chardonnay’ and ‘Grillo’ cultivar grown on limestone and marlstone soils in 2021 and 2022 vintages. Table S11. Flavonols content during ripening of ‘Chardonnay’ and ‘Grillo’ cultivar grown on limestone and marlstone soils in 2021 and 2022 vintages. Table S12. Hydrocycinnamoyltartaric acids (HCTAs) content during ripening of ‘Chardonnay’ and ‘Grillo’ cultivar grown on limestone and marlstone soils in 2021 and 2022 vintages. Table S13. Technological parameters content during ripening of ‘Nero d’Avola’ and ‘Syrah’ cultivar grown on limestone and marlstone soils in 2021 and 2022 vintages. Table S14. Skin and seeds total flavonoids and anthocyanins content during ripening of ‘Nero d’Avola’ and ‘Syrah’ cultivar grown on limestone and marlstone soils in 2021 and 2022 vintages. Table S15. Flavonols content during ripening of ‘Nero d’Avola’ and ‘Syrah’ cultivar [file JSFA-106-1079-s001.docx]

Unveiling grape ripening heterogeneity in white and red cultivars (*Vitis vinifera* L.) grown on different calcareous soils using technological and phenolic markers

Clara Vitaggio^1*^, Matteo Pollon^1^, Manuel Schnitter^1^,

Luciano Cinquanta^1^, Onofrio Corona^1^

^1^ Department of Agricultural, Food and Forest Sciences, University of Palermo, (Palermo), Viale delle Scienze 13, 90128 Palermo, Italy

[matteo.pollon@unipa.it](mailto:matteo.pollon@unipa.it); [manuel.chnitter@unipa.it](mailto:manuel.chnitter@unipa.it); [valentina.caraci@unipa.it](mailto:valentina.caraci@unipa.it); [luciano.cinquanta@unipa.it](mailto:luciano.cinquanta@unipa.it); [onofrio.corona@unipa.it](mailto:onofrio.corona@unipa.it).

*corresponding author: [clara.vitaggio@unipa.it](mailto:clara.vitaggio@unipa.it)


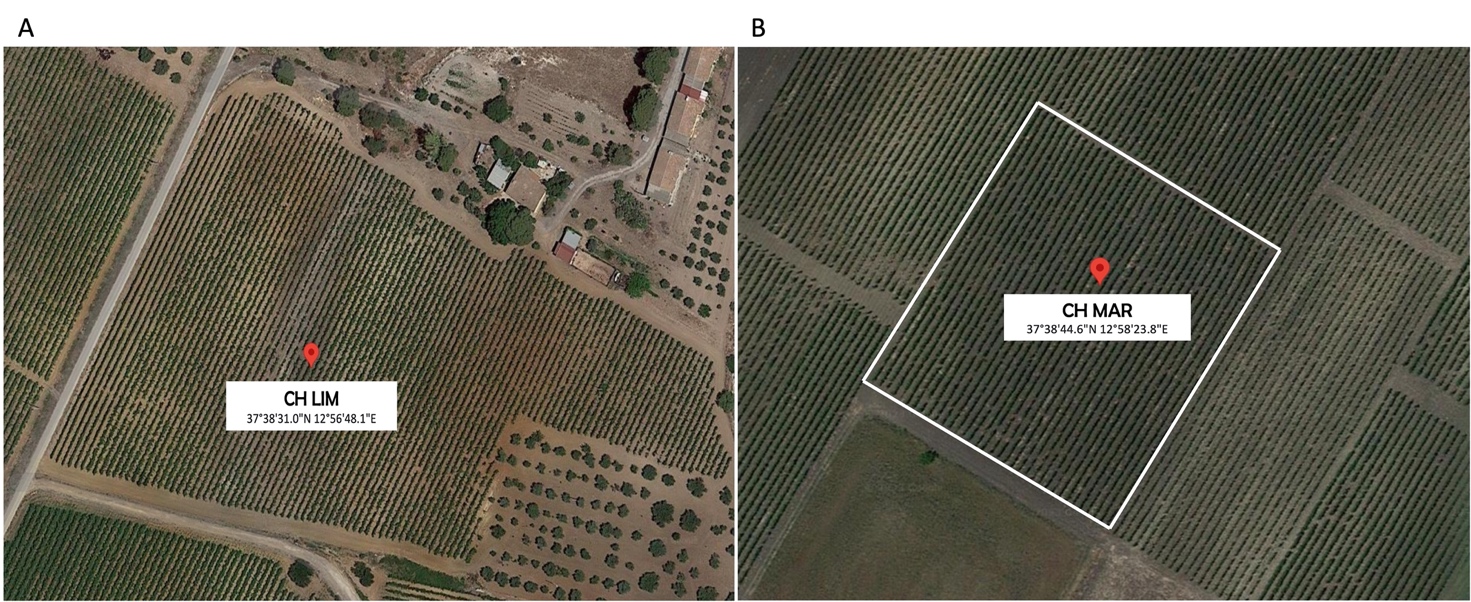


Figure S1 Satellite images of the two vineyard parcels used in the study for Chardonnay cultivar: limestone soil (CH LIM) (A) and marlstone soil (CH MAR) (B), both located in southwestern Sicily. The coordinates of each vineyard are indicated.


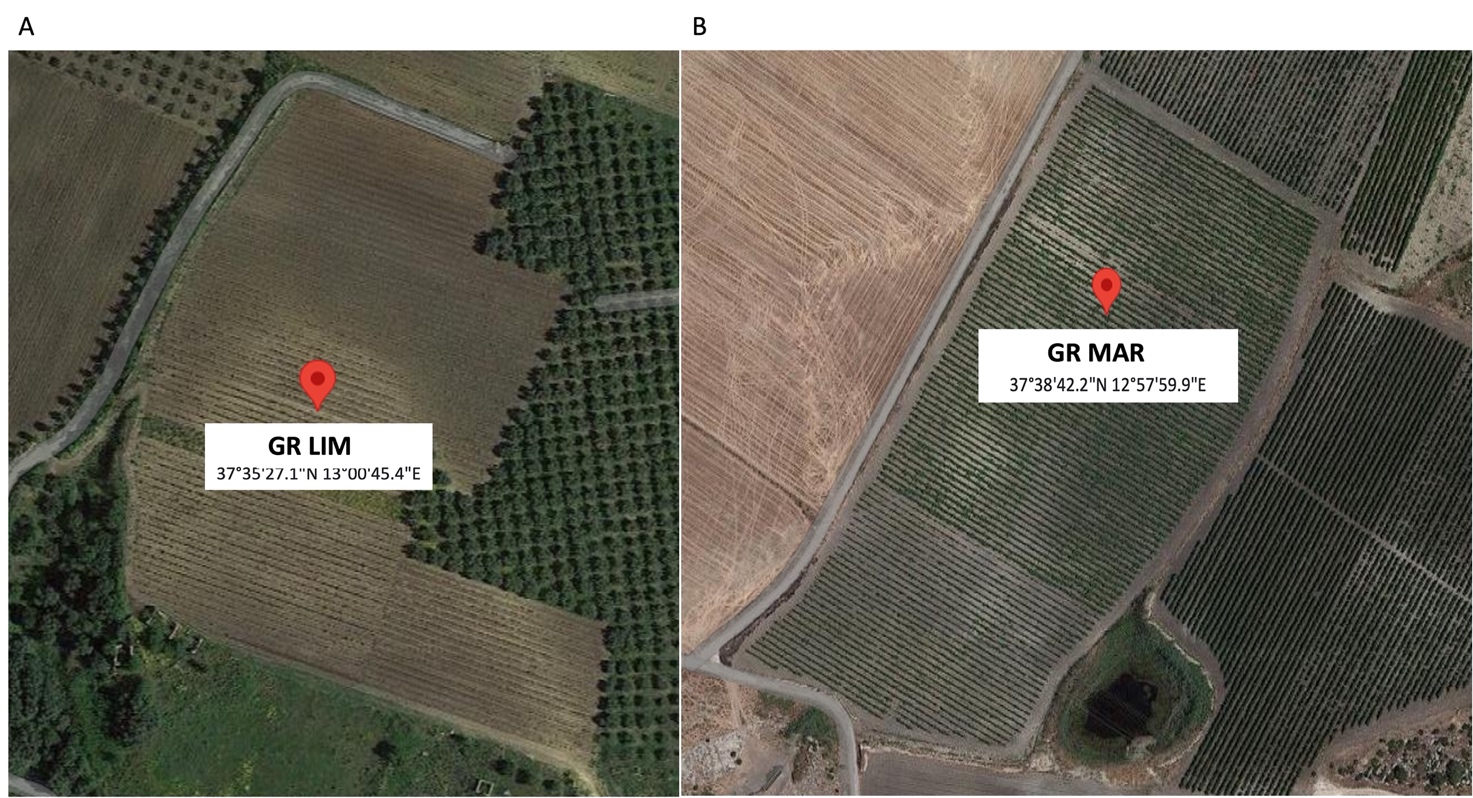


Figure S2 Satellite images of the two vineyard parcels used in the study for Grillo cultivar: limestone soil (GR LIM) (A) and marlstone soil (GR MAR) (B), both located in southwestern Sicily. The coordinates of each vineyard are indicated.


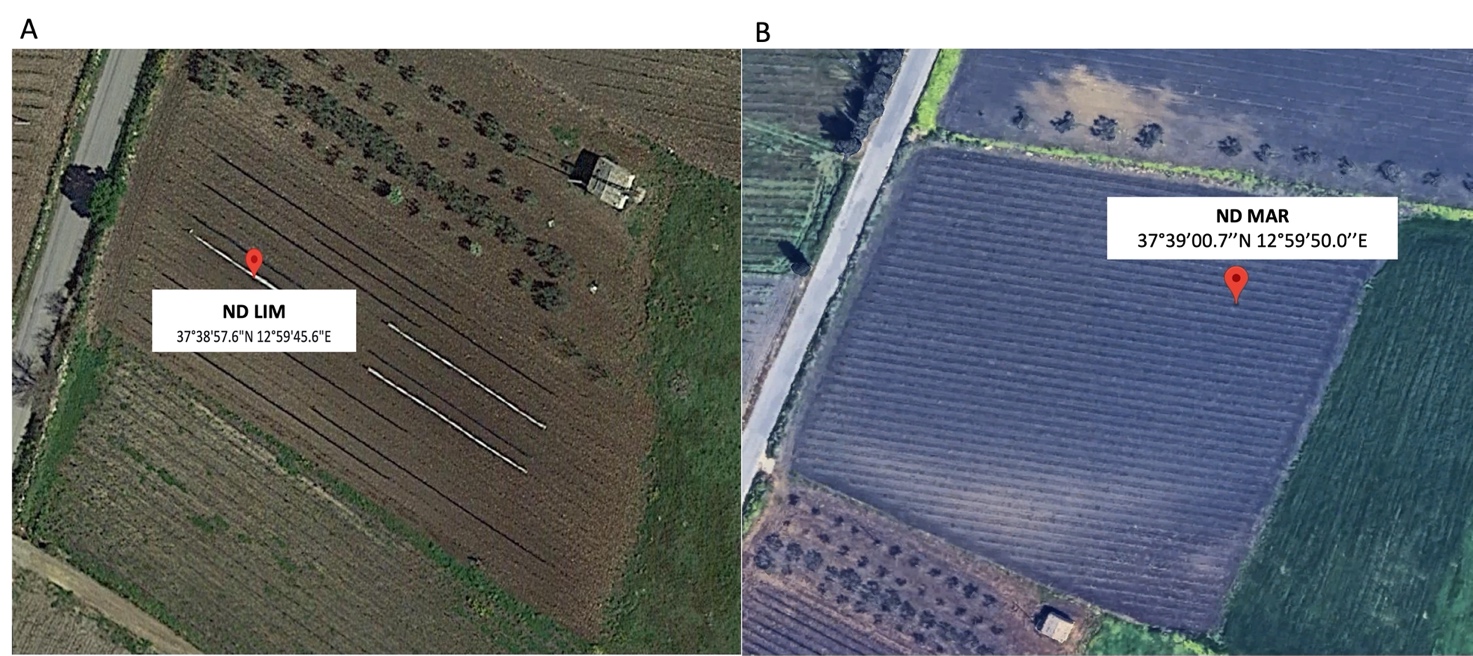


Figure S3 Satellite images of the two vineyard parcels used in the study for Nero d’Avola cultivar: limestone soil (ND LIM) (A) and marlstone soil (ND MAR) (B), both located in southwestern Sicily. The coordinates of each vineyard are indicated.


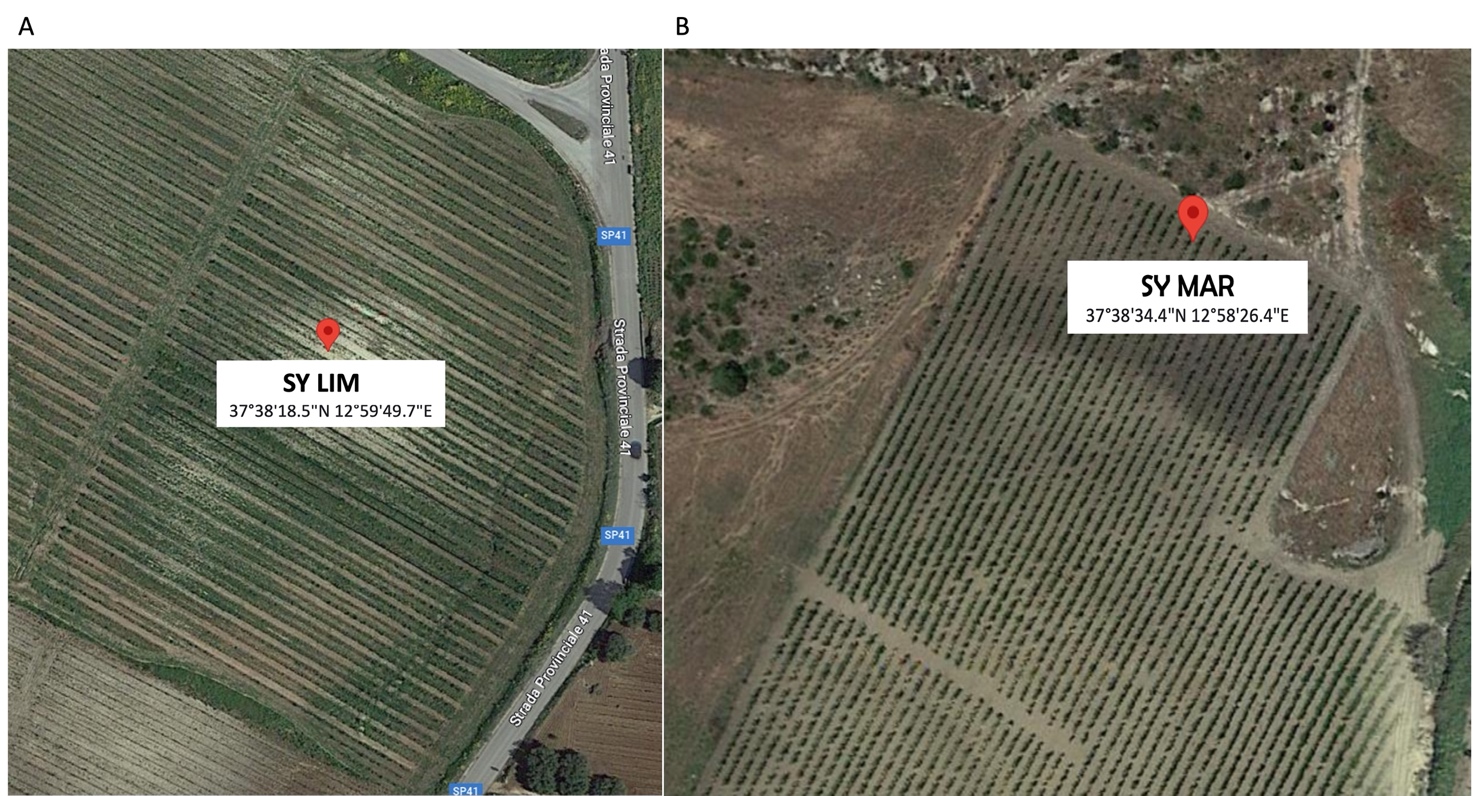


Figure S4 Satellite images of the two vineyard parcels used in the study for Syrah cultivar: limestone soil (SY LIM) (A) and marlstone soil (SY MAR) (B), both located in southwestern Sicily. The coordinates of each vineyard are indicated.

Table S1 Chemical and physical parameters of the limestone soil (CH LIM) where Chardonnay vines are cultivated.

| **CH LIM** | | | | | | |
| --- | --- | --- | --- | --- | --- | --- |
| Soil depth (cm) | Total limestone (%) | Organic matter (%) | Active lime (%) | Sand (%) | Clay (%) | Silt (%) |
| 20 | 13.7 | 1.3 | 2 | 61 | 31 | 8 |
| 40 | 10.2 | 1.11 | 1.25 | / | / | / |
| 60 | 18 | 0.73 | 2.75 | / | / | / |
| 80 | 28.2 | 0.31 | 8.75 | / | / | / |

Table S2 Chemical and physical parameters of the marlstone soil (CH MAR) where Chardonnay vines are cultivated.

| **CH MAR** | | | | | | |
| --- | --- | --- | --- | --- | --- | --- |
| Soil depth (cm) | Total limestone (%) | Organic matter (%) | Active lime (%) | Sand (%) | Clay (%) | Silt (%) |
| 20 | 24.1 | 3.69 | 12.75 | 35 | 36 | 29 |
| 40 | 24.5 | 2.89 | 16.25 | / | / | / |
| 60 | 24.5 | 2.18 | 14.25 | / | / | / |
| 80 | 24.9 | 1.72 | 14.75 | / | / | / |
| 100 | 24.1 | 1.68 | 14.75 | / | / | / |

Table S3 Chemical and physical parameters of the limestone soil (GR LIM) where Grillo vines are cultivated.

| **GR LIM** | | | | | | |
| --- | --- | --- | --- | --- | --- | --- |
| Soil depth (cm) | Total limestone (%) | Organic matter (%) | Active lime (%) | Sand (%) | Clay (%) | Silt (%) |
| 20 | 51.1 | 2.02 | 17.25 | 59 | 21 | 20 |
| 40 | 56.9 | 1.52 | 17.25 | / | / | / |
| 60 | 48.6 | 2.28 | 19.75 | / | / | / |
| 80 | 61.9 | 0.61 | 20.15 | / | / | / |

Table S4 Chemical and physical parameters of the marlstone soil (GR MAR) where Grillo vines are cultivated.

| **GR MAR** | | | | | | |
| --- | --- | --- | --- | --- | --- | --- |
| Soil depth (cm) | Total limestone (%) | Organic matter (%) | Active lime (%) | Sand (%) | Clay (%) | Silt (%) |
| 20 | 38.2 | 1.72 | 10 | 18 | 51 | 31 |
| 40 | 37.4 | 1.3 | 15 | / | / | / |
| 60 | 40.3 | 0.96 | 15.75 | / | / | / |
| 80 | 41.1 | 0.57 | 16 | / | / | / |
| 100 | 41.5 | 1.72 | 16.2 | / | / | / |

Table S5 Chemical and physical parameters of the limestone soil (ND LIM) where Nero d’Avola vines are cultivated.

| **ND LIM** | | | | | | |
| --- | --- | --- | --- | --- | --- | --- |
| Soil depth (cm) | Total limestone (%) | Organic matter (%) | Active lime (%) | Sand (%) | Clay (%) | Silt (%) |
| 20 | 20.7 | 3.1 | 4 | 28.1 | 28.5 | 43.4 |
| 40 | 23.7 | 3.3 | 4.3 | / | / | / |
| 60 | 18 | 3.4 | 4.2 | / | / | / |

Table S6 Chemical and physical parameters of the marlstone soil (ND MAR) where Nero d’Avola vines are cultivated.

| **ND MAR** | | | | | | |
| --- | --- | --- | --- | --- | --- | --- |
| Soil depth (cm) | Total limestone (%) | Organic matter (%) | Active lime (%) | Sand (%) | Clay (%) | Silt (%) |
| 20 | 17.9 | 2.9 | 9.8 | 20.96 | 43.5 | 35..54 |
| 40 | 16.6 | 2.1 | 8.3 | / | / | / |
| 60 | 17.4 | 1.9 | 9.6 | / | / | / |
| 80 | 18.7 | 2.3 | 9.9 | / | / | / |

Table S7 Chemical and physical parameters of the limestone soil (SY LIM) where Syrah vines are cultivated.

| **SY LIM** | | | | | | |
| --- | --- | --- | --- | --- | --- | --- |
| Soil depth (cm) | Total limestone (%) | Organic matter (%) | Active lime (%) | Sand (%) | Clay (%) | Silt (%) |
| 20 | 20 | 4.4 | 3 | 34.4 | 38.5 | 27.1 |
| 40 | 21.1 | 3.9 | 7.8 | / | / | / |
| 60 | 22.1 | 3.7 | 5 | / | / | / |

Table S8 Chemical and physical parameters of the marlstone soil (SY MAR) where Syrah vines are cultivated.

| **SY MAR** | | | | | | |
| --- | --- | --- | --- | --- | --- | --- |
| Soil depth (cm) | Total limestone (%) | Organic matter (%) | Active lime (%) | Sand (%) | Clay (%) | Silt (%) |
| 20 | 56.1 | 2.5 | 14.75 | 34.4 | 46 | 20 |
| 40 | 59.8 | 1.37 | 15.25 | / | / | / |
| 60 | 66 | 0.84 | 18.75 | / | / | / |
| 80 | 69.8 | 0.08 | 21 | / | / | / |
| 100 | 60.2 | 0.42 | 19.75 | / | / | / |

Table S9 Technological parameters content during ripening of Chardonnay and Grillo cultivar grown on limestone and marlstone soils in 2021 and 2022 vintages.

| **Vintage 2021** | | | | | |
| --- | --- | --- | --- | --- | --- |
| **CH LIM** | | | | | |
|  | **Density class** | **% m/m** | **Reducing sugars g L^-1^** | **Titratable acidity g L^-1^** | **pH** |
| I | 1071 | 12 | 148 ± 5 | 7 ± 1 | 3.48 ± 0.01 |
| II | 1075 | 24 | 160 ± 10 | 5.0 ± 0.3 | 3.49 ± 0.02 |
| III | 1088 | 9 | 173 ± 8 | 4.8 ± 0.4 | 3.54 ± 0.03 |
| IV | 1101 | 15 | 204 ± 7 | 4.3 ± 0.5 | 3.53 ± 0.02 |
| V | 1126 | 39 | 220 ± 4 | 4.4 ± 0.2 | 3.52 ± 0.03 |
| **CH MAR** | | | | | |
| I | 1075 | 8 | 212 ± 4 | 8 ± 1 | 3.73 ± 0.02 |
| II | 1088 | 25 | 225 ± 3 | 8 ± 1 | 3.68 ± 0.02 |
| III | 1101 | 37 | 241 ± 9 | 8 ± 1 | 3.65 ± 0.02 |
| IV | 1113 | 20 | 248 ± 7 | 7 ± 1 | 3.71 ± 0.00 |
| V | 1126 | 4 | 271 ± 8 | 6.9 ± 0.5 | 3.77 ± 0.01 |
| **GR LIM** | | | | | |
| I | 1075 | 23 | 154 ± 3 | 8 ± 1 | 2.96 ± 0.00 |
| II | 1088 | 28 | 194 ± 10 | 7.1 ± 0.2 | 3.10 ± 0.02 |
| III | 1101 | 11 | 222 ± 9 | 7.0 ± 0.2 | 3.13 ± 0.01 |
| IV | 1113 | 15 | 250 ± 4 | 7.8 ± 0.1 | 3.09 ± 0.02 |
| V | 1088 | **/** | 279 ± 4 | 7.4 ± 0.3 | 3.22 ± 0.03 |
| **GR MAR** | | | | | |
| I | 1075 | **/** | **/** | **/** | **/** |
| II | 1088 | 36 | 226 ± 5 | 8.4 ± 0.3 | 3.27 ± 0.02 |
| III | 1101 | 35 | 254 ± 6 | 8.8 ± 1.1 | 3.17 ± 0.03 |
| IV | 1113 | 23 | 287 ± 6 | 8.1 ± 0.3 | 3.31 ± 0.01 |
| V | 1126 | 5 | 331 ± 5 | 7.7 ± 0.3 | 3.35 ± 0.01 |
|  |  |  |  |  |  |
| **Vintage 2022** | | | | | |
| **CH LIM** | | | | | |
|  | **Density class** | **% m/m** | **Reducing sugars g L^-1^** | **Titratable acidity g L^-1^** | **pH** |
| I | 1075 | 17 | 173 ± 7 | 7.1 ± 0.5 | 3.27 ± 0.01 |
| II | 1088 | 28 | 203 ± 4 | 6.9 ± 0.2 | 3.30 ± 0.01 |
| III | 1101 | 28 | 231 ± 5 | 7 ± 1 | 3.21 ± 0.03 |
| IV | 1113 | 17 | 257 ± 5 | 6.1 ± 0.2 | 3.30 ± 0.03 |
| V | 1126 | 7 | 303 ± 6 | 6 ± 1 | 3.46 ± 0.02 |
| **CH MAR** | | | | | |
| I | 1075 | 13 | 176 ± 8 | 6 ± 1 | 3.56 ± 0.03 |
| II | 1088 | 33 | 192 ± 4 | 6.2 ± 0.2 | 3.67 ± 0.03 |
| III | 1101 | 39 | 214 ± 6 | 6.2 ± 0.1 | 3.66 ± 0.01 |
| IV | 1113 | 12 | 233 ± 7 | 6 ± 1 | 3.70 ± 0.03 |
| V | 1126 | 2 | 261 ± 3 | 6 ± 1 | 3.73 ± 0.02 |
| **GR LIM** | | | | | |
| I | 1075 | 11 | 173 ± 7 | 8 ± 1 | 3.08 ± 0.03 |
| II | 1088 | 24 | 213 ± 4 | 8 ± 1 | 3.10 ± 0.03 |
| III | 1101 | 15 | 239 ± 3 | 7.9 ± 0.2 | 3.09 ± 0.01 |
| IV | 1113 | 40 | 246 ± 10 | 7.7 ± 0.3 | 3.12 ± 0.01 |
| V | 1126 | 4 | 294 ± 10 | 7.6 ± 0.2 | 3.18 ± 0.01 |
| **GR MAR** | | | | | |
| I | 1075 | 3 | 203 ± 5 | 7 ± 1 | 3.24 ± 0.03 |
| II | 1088 | 9 | 224 ± 3 | 6.4 ± 0.3 | 3.29 ± 0.03 |
| III | 1101 | 39 | 253 ± 8 | 6.4 ± 0.1 | 3.42 ± 0.00 |
| IV | 1113 | 27 | 277 ± 6 | 6.2 ± 0.3 | 3.45 ± 0.02 |
| V | 1126 | 22 | 311 ± 3 | 6.2 ± 0.3 | 3.43 ± 0.02 |

Note: Data are reported as mean plus and minus standard error of the mean. CH LIM = *Chardonnay* on limestone; CH MAR = *Chardonnay* on marlstone; GR LIM = *Grillo* on limestone; GR MAR = *Grillo* on marlstone.

Table S10 Skin and seeds total flavonoids content during ripening of Chardonnay and Grillo cultivar grown on limestone and marlstone soils in 2021 and 2022 vintages.

|  | **Vintage 2021** | | | | |
| --- | --- | --- | --- | --- | --- |
|  | **CH LIM** | | | | |
|  | | **Density class** | **% m/m** | **Skins total flavonoids (mg 100 berries^-1^)** | **Seeds total flavonoids (mg 100 berries^-1^)** |
| I | | 1071 | 12 | 74 ± 6 | 331 ± 84 |
| II | | 1075 | 24 | 73 ± 6 | 397 ± 23 |
| III | | 1088 | 9 | 66 ± 6 | 314 ± 33 |
| IV | | 1101 | 15 | 87 ± 6 | 312 ± 2 |
| V | | 1113 | 39 | 83 ± 11 | 313 ± 14 |
| VI | | 1126 | / | / | / |
|  | **CH MAR** | | | | |
|  | | **Density class** | **% m/m** | **Skins total flavonoids (mg 100 berries^-1^)** | **Seeds total flavonoids (mg 100 berries^-1^)** |
| I | | 1071 | 8 | 61 ± 4 | 334 ± 7 ab |
| II | | 1075 | 25 | 61 ± 5 | 379 ± 7 a |
| III | | 1088 | 37 | 56 ± 1 | 264 ± 10 ab |
| IV | | 1101 | 20 | 59 ± 4 | 222 ± 74 b |
| V | | 1113 | 4 | 63 ± 8 | 262 ± 32 ab |
| VI | | 1126 | / | / | / |
|  | **GR LIM** | | | | |
|  | | **Density class** | **% m/m** | **Skins total flavonoids (mg 100 berries^-1^)** | **Seeds total flavonoids (mg 100 berries^-1^)** |
| I | | 1071 | 18 | 108 ± 2 | 447 ± 8 |
| II | | 1075 | 29 | 110 ± 14 | 443 ± 89 |
| III | | 1088 | 11 | 99 ± 17 | 379 ± 52 |
| IV | | 1101 | 15 | 98 ± 7 | 454 ± 44 |
| V | | 1113 | / | / | / |
| VI | | 1126 | / | / | / |
|  | **GR MAR** | | | | |
|  | | **Density class** | **% m/m** | **Skins total flavonoids (mg 100 berries^-1^)** | **Seeds total flavonoids (mg 100 berries^-1^)** |
| I | | 1071 | / | / | / |
| II | | 1075 | / | / | / |
| III | | 1088 | 36 | 121 ± 14 | 510 ± 74 |
| IV | | 1101 | 35 | 109 ± 9 | 422 ± 100 |
| V | | 1113 | 23 | 120 ± 8 | 435 ± 37 |
| VI | | 1126 | 5 | 86 ± 7 | 365 ± 96 |
|  | |  |  |  |  |
|  | |  |  |  |  |
|  | **Vintage 2022** | | | | |
|  | **CH LIM** | | | | |
|  | | **Density class** | **% m/m** | **Skins total flavonoids (mg 100 berries^-1^)** | **Seeds total flavonoids (mg 100 berries^-1^)** |
| I | | 1071 | 4 | 94 ± 8 | 509 ± 6 |
| II | | 1075 | 17 | 91 ± 8 | 559 ± 4 |
| III | | 1088 | 28 | 81 ± 7 | 416 ± 1 |
| IV | | 1101 | 28 | 111 ± 5 | 473 ± 1 |
| V | | 1113 | 17 | 122 ± 6 | 334 ± 51 |
| VI | | 1126 | 7 | 82 ± 2 | 300 ± 19 |
|  | **CH MAR** | | | | |
|  | | **Density class** | **% m/m** | **Skins total flavonoids (mg 100 berries^-1^)** | **Seeds total flavonoids (mg 100 berries^-1^)** |
| I | | 1071 | / | / | / |
| II | | 1075 | 13 | 143 ± 16 ab | 610 ± 72 a |
| III | | 1088 | 33 | 145 ± 4 ab | 445 ± 21 ab |
| IV | | 1101 | 39 | 175 ± 7 a | 351 ± 87 bc |
| V | | 1113 | 12 | 119 ± 14 bc | 348 ± 25 bc |
| VI | | 1126 | 2 | 81 ± 19 c | 217 ± 23 c |
|  | **GR LIM** | | | | |
|  | | **Density class** | **% m/m** | **Skins total flavonoids (mg 100 berries^-1^)** | **Seeds total flavonoids (mg 100 berries^-1^)** |
| I | | 1071 | 6 | 133 ± 29 | 625 ± 49 a |
| II | | 1075 | 11 | 131 ± 11 | 614 ± 21 a |
| III | | 1088 | 24 | 167 ± 22 | 636 ± 3 a |
| IV | | 1101 | 15 | 162 ± 45 | 541 ± 33 ab |
| V | | 1113 | 40 | 181 ± 7 | 662 ± 94 a |
| VI | | 1126 | 4 | 117 ± 7 | 359 ± 39 b |
|  | **GR MAR** | | | | |
|  | | **Density class** | **% m/m** | **Skins total flavonoids (mg 100 berries^-1^)** | **Seeds total flavonoids (mg 100 berries^-1^)** |
| I | | 1071 | / | / | / |
| II | | 1075 | 3 | 158 ± 8 ab | 534 ± 32 |
| III | | 1088 | 9 | 178 ± 3 a | 510 ± 74 |
| IV | | 1101 | 39 | 142 ± 14 b | 464 ± 41 |
| V | | 1113 | 27 | 157 ± 2 ab | 435 ± 37 |
| VI | | 1126 | 22 | 131 ± 2 b | 447 ± 21 |

Note: Data are reported as mean plus and minus standard error of the mean. Sign. =ANOVA. Different letters indicate statistically significant differences according to post-hoc tests (*p* < 0.05). CH LIM = *Chardonnay* on limestone; CH MAR = *Chardonnay* on marlstone; GR LIM = *Grillo* on limestone; GR MAR = *Grillo* on marlstone.

Table S11 Flavonols content (g kg^-1^ of berries) during ripening of Chardonnay and Grillo cultivar grown on limestone and marlstone soils in 2021 and 2022 vintages.

| **Vintage 2021** | | | | | | | |
| --- | --- | --- | --- | --- | --- | --- | --- |
| **CH LIM** | | | | | | | |
| **Density class** | **% m/m** | **Quercetin 3-glucuronide** | **Quercetin 3-glucoside** | **Laricitrin-3-glucoside** | **kaempferol-3-glucuronide** | **kaempferol-3-glucoside** | **Quercetin aglicon** |
| I | 12 | 2.6 ± 0.3 a | 194 ± 9 a | 132 ± 29 ab | 3 ± 1 b | 15 ± 6 a | 2.0 ± 0.4 a |
| II | 24 | 3.0 ± 0.5 a | 213 ± 45 a | 161 ± 26 a | 4 ± 1 a | 23 ± 4 a | 2.7 ± 0.2 a |
| III | 9 | 0.6 ± 0.1 b | 64 ± 3 b | 61 ± 6 bc | 3 ± 1 b | 14 ± 2 ab | 0.8 ± 0.1 b |
| IV | 15 | 0.2 ± 0.2 | 2 ± 2 b | 2 ± 1 c | BDL | 0.2 ± 0.2 c | BDL |
| V | 39 | BDL | 0.3 ± 0.1 b | 0.9 ± 0.4 c | BDL | 0.29 ± 0.04 bc | BDL |
| VI | / | / | / | / | / | / | / |
| **CH MAR** | | | | | | | |
| I | / | / | / | / | / | / | / |
| II | 8 | 1.7 ± 0.4 a | 107 ± 48 | 97 ± 28 a | 5 ± 3 | 27 ± 13 | 8 ± 4 |
| III | 25 | 0.66 ± 0.03 b | 21.8 ± 0.4 | 22.0 ± 0.3 b | 1.25 ± 0.03 | 6.5 ± 0.3 | 0.9 ± 0.1 |
| IV | 37 | 0.37 ± 0.01 b | 26 ± 1 | 25 ± 1 b | 1.23 ± 0.02 | 5.9 ± 0.3 | 1.98 ± 0.01 |
| V | 20 | 0.6 ± 0.4 b | 37 ± 3 | 37 ± 11 b | 1.9 ± 1.1 | 9 ± 4 | 2 ± 1 |
| VI | 4 | 0.5 ± 0.1 b | 29 ± 4 | 36 ± 4 b | 2.4 ± 0.2 | 11 ± 1 | 1.2 ± 0.3 |
|  |  |  |  |  |  |  |  |
| **Vintage 2021** | | | | | | | |
| **GR LIM** | | | | | | | |
| I | 18 | 1 ± 1 | 108 ± 74 | 69 ± 58 | 4 ± 4 | 15 ± 18 | 3 ± 3 |
| II | 29 | 0.4 ± 0.3 | 29 ± 36 | 13 ± 18 | 0.4 ± 0.6 | 2 ± 3 | 1 ± 1 |
| III | 11 | 0.30 ± 0.01 | 30.1 ± 0.2 | 17 ± 1 | 0.6 ± 0.2 | 3 ± 1 | 0.8 ± 0.1 |
| IV | 15 | 0.3 ± 0.1 | 28 ± 2 | 21 ± 5 | 1 ± 1 | 7 ± 3 | 0.8 ± 0.1 |
| V | / | / | / | / | / | / | / |
| VI | / | / | / | / | / | / | / |
| **GR MAR** | | | | | | | |
| I | / | / | / | / | / | / | / |
| II | / | / | / | / | / | / | / |
| III | 36 | 0.4 ± 0.2 | 38 ± 14 | 28 ± 18 | 2 ± 2 | 9 ± 8 | 1.2 ± 0.3 |
| IV | 35 | 0.55 ± 0.01 | 59 ± 3 | 46 ± 8 | 4 ± 1 | 18 ± 6 | 1.80 ± 0.04 |
| V | 23 | 0.3 ± 0.1 | 60 ± 2 | 51 ± 5 | 4.6 ± 0.3 | 22 ± 3 | 1.5 ± 0.2 |
| VI | 5 | 0.21 ± 0.04 | 41 ± 5 | 29 ± 4 | 3 ± 1 | 14 ± 3 | 1.3 ± 0.2 |
|  |  |  |  |  |  |  |  |
| **Vintage 2022** | | | | | | | |
| **CH LIM** | | | | | | | |
| **Density class** | **% m/m** | **Quercetin 3-glucuronide** | **Quercetin 3-glucoside** | **Laricitrin-3-glucoside** | **kaempferol-3-glucuronide** | **kaempferol-3-glucoside** | **Quercetin aglicon** |
| I | 4 | 104 ± 9 a | 59 ± 15 ab | / | 0.3 ± 0.5 c | 6 ± 2 | / |
| II | 17 | 100 ± 18 a | 64 ± 9 a | / | 1.7 ± 0.2 ab | 8 ± 1 | / |
| III | 28 | 36 ± 2 b | 28 ± 2 bc | / | 1.0 ± 0.1 bc | 5.8 ± 0.4 | / |
| IV | 28 | 33 ± 1 b | 23 ± 2 c | / | 0.8 ± 0.3 bc | 4 ± 1 | / |
| V | 17 | 15 ± 0 b | 13.6 ± 0.1 c | / | 0.50 ± 0.01 c | 2.36 ± 0.01 | / |
| VI | 7 | 34 ± 2 b | 37 ± 9 abc | / | 2.2 ± 0.3 a | 12 ± 5 | / |
| **CH MAR** | | | | | | | |
| I | / | 64 ± 2 | 32 ± 1 b | 0.61 ± 0.02 | 0.73 ± 0.03 b | 6.4 ± 0.3 b | / |
| II | 13 | 83 ± 35 | 46 ± 19 b | 2 ± 1 | 2 ± 1 b | 9 ± 4 b | / |
| III | 33 | 42 ± 7 | 24 ± 6 b | 0.6 ± 0.1 | 0.9 ± 0.2 b | 5 ± 2 b | / |
| IV | 39 | 57 ± 9 | 37 ± 10 b | 0.9 ± 0.3 | 2 ± 1 b | 8 ± 4 b | / |
| V | 12 | 73 ± 8 | 65 ± 19 ab | 1 ± 1 | 4 ± 2 b | 20 ± 10 ab | / |
| VI | 2 | 103 ± 12 | 105 ± 15 a | BDL | 11 ± 2 a | 45 ± 12 a | / |
| **GR LIM** | | | | | | | |
| I | 6 | 20 ± 9 abc | 6 ± 2 c | / | BDL | 1.1 ± 0.3 cd | / |
| II | 11 | 29.56 ± 0.02 ab | 17 ± 2 b | / | 0.52 ± 0.05 c | 3.6 ± 0.1 b | / |
| III | 24 | 8 ± 2 c | 4 ± 1 c | / | 0.11 ± 0.03 ab | 0.7 ± 0.3 d | / |
| IV | 15 | 9.8 ± 0.5 c | 7 ± 3 c | / | 0.2 ± 0.1 ab | 1 ± 1 cd | / |
| V | 40 | 13 ± 1 bc | 12 ± 1 bc | / | 0.5 ± 0.1 c | 2.8 ± 0.3 bc | / |
| VI | 4 | 35 ± 5 a | 31 ± 4 a | / | 1.9 ± 0.2 a | 10 ± 1 a | / |
| **GR MAR** | | | | | | | |
| I | / | / | / | / | / | / | / |
| II | 3 | 161.22 ± 0.01 a | 123 ± 1 b | 4 ± 2 | 5.81 ± 0.05 a | 35 ± 1 a | / |
| III | 9 | 33 ± 4 bc | 23 ± 6 a | 0.6 ± 0.2 | 1.1 ± 0.2 b | 7 ± 2 b | / |
| IV | 39 | 31 ± 3 bc | 21 ± 5 b | 0.38 ± 0.01 | 0.8 ± 0.2 b | 7 ± 2 b | / |
| V | 27 | 40 ± 2 b | 29 ± 1 b | 0.49 ± 0.01 | 1.37 ± 0.05 b | 10 ± 1 b | / |
| VI | 22 | 27 ± 3 c | 18 ± 1 b | 0.4 ± 0.1 | 1.0 ± 0.1 b | 7 ± 1 b | / |

Note: Data are reported as mean plus and minus standard error of the mean. Sign. =ANOVA. Different letters indicate statistically significant differences according to post-hoc tests (*p* < 0.05). CH LIM = *Chardonnay* on limestone; CH MAR = *Chardonnay* on marlstone; GR LIM = *Grillo* on limestone; GR MAR = *Grillo* on marlstone. Density class: I= 1071, II = 1075, III = 1088, IV = 1101, V = 1113, VI = 1126 kg m^-3^.

Table S12 Hydrocycinnamoyltartaric acids (HCTAs) content (g kg^-1^ of berries) during ripening of Chardonnay and Grillo cultivar grown on limestone and marlstone soils in 2021 and 2022 vintages.

| **Vintage 2021** | | | | | |
| --- | --- | --- | --- | --- | --- |
| **CH LIM** | | | | | |
|  | **Density class** | **% m/m** | **Caftaric acid** | **Coutaric acid** | **Feftaric acid** |
| I | 1071 | 12 | 1356 ± 155 a | 1038 ± 103 a | 38 ± 21 b |
| II | 1075 | 24 | 1681 ± 158 a | 1150 ± 74 a | 75 ± 11 a |
| III | 1088 | 9 | 229 ± 23 b | 204 ± 13 b | 11 ± 2 c |
| IV | 1101 | 15 | 213 ± 113 b | 110 ± 78 b | 5 ± 3 c |
| V | 1126 | 39 | 17 ± 9 c | 13 ± 4 c | 4 ± 2 c |
| **CH MAR** | | | | | |
| I | 1075 | 8 | 1330 ± 149 a | 657 ± 33 a | 74 ± 13 a |
| II | 1088 | 25 | 290 ± 5 b | 83 ± 81 b | 7 ± 6 b |
| III | 1101 | 37 | 113 ± 108 b | 81 ± 77 b | 11 ± 1 b |
| IV | 1113 | 20 | 226 ± 12 b | 134 ± 8 b | 21 ± 3 b |
| V | 1126 | 4 | 155 ± 13 b | 100 ± 10 b | 11 ± 1 b |
| **GR LIM** | | | | | |
| I | 1075 | 23 | 620 ± 159 | 219 ± 59 | 54 ± 20 |
| II | 1088 | 28 | 316 ± 29 | 111 ± 8 | 24 ± 2 |
| III | 1101 | 11 | 205 ± 12 | 74 ± 5 | 17 ± 1 |
| IV | 1113 | 15 | 150 ± 16 | 57 ± 3 | 16 ± 2 |
| V | 1126 | **/** | **/** | **/** | **/** |
| **GR MAR** | | | | | |
| I | 1075 | **/** | **/** | **/** | **/** |
| II | 1088 | 36 | 164 ± 14 | 63 ± 9 | 17 ± 2 |
| III | 1101 | 35 | 211 ± 13 | 86 ± 7 | 21 ± 1 |
| IV | 1113 | 23 | 198 ± 16 | 84 ± 3 | 17 ± 5 |
| V | 1126 | 5 | 114 ± 1 | 48 ± 2 | 15 ± 5 |
|  |  |  |  |  |  |
| **Vintage 2022** | | | | | |
| **CH LIM** | | | | | |
|  | **Density class** | **% m/m** | **Caftaric acid** | **Coutaric acid** | **Feftaric acid** |
| II | 1075 | 17 | 923 ± 37 a | 629 ± 17 a | 30 ± 7 |
| III | 1088 | 28 | 170 ± 9 c | 142 ± 7 c | 9 ± 1 |
| IV | 1101 | 28 | 249 ± 5 c | 188 ± 4 c | 10 ± 1 |
| V | 1113 | 17 | 133 ± 4 c | 87 ± 3 b | 11 ± 1 |
| VI | 1126 | 7 | 279 ± 12 b | 175 ± 4 c | 17 ± 1 |
| **CH MAR** | | | | | |
| II | 1075 | 13 | 282 ± 98 | 129 ± 44 | 23 ± 11 |
| III | 1088 | 33 | 175 ± 39 | 73 ± 13 | 10 ± 2 |
| IV | 1101 | 39 | 241 ± 8 | 99 ± 5 | 13 ± 1 |
| V | 1113 | 12 | 261 ± 13 | 105 ± 5 | 29 ± 6 |
| VI | 1126 | 2 | 301 ± 42 | 86 ± 43 | 84 ± 51 |
| **GR LIM** | | | | | |
| II | 1075 | 11 | 382 ± 28 | 136 ± 1 | 17 ± 1 |
| III | 1088 | 24 | 101 ± 1 | 36 ± 1 | 4 ± 1 |
| IV | 1101 | 15 | 103 ± 11 | 37 ± 2 | 5 ± 1 |
| V | 1113 | 40 | 115 ± 3 | 43 ± 1 | 6 ± 1 |
| VI | 1126 | 4 | 130 ± 3 | 57 ± 1 | 10 ± 1 |
| **GR MAR** | | | | | |
| II | 1075 | 3 | 668 ± 29 | 271 ± 11 | 48 ± 10 |
| III | 1088 | 9 | 145 ± 3 | 58 ± 3 | 12 ± 1 |
| IV | 1101 | 39 | 126 ± 14 | 50 ± 6 | 10 ± 1 |
| V | 1113 | 27 | 151 ± 7 | 60 ± 4 | 13 ± 1 |
| VI | 1126 | 22 | 83 ± 1 | 31 ± 1 | 10 ± 1 |

Note: Data are reported as mean plus and minus standard error of the mean. Sign. =ANOVA. Different letters indicate statistically significant differences according to post-hoc tests (*p* < 0.05). CH LIM = *Chardonnay* on limestone; CH MAR = *Chardonnay* on marlstone; GR LIM = *Grillo* on limestone; GR MAR = *Grillo* on marlstone.

Table S13 Technological parameters content during ripening of Nero d’Avola and Syrah cultivar grown on limestone and marlstone soils in 2021 and 2022 vintages.

| **Vintage 2021** | | | | | |
| --- | --- | --- | --- | --- | --- |
| **ND LIM** | | | | | |
|  | **Density class** | **% m/m** | **Reducing sugars g L^-1^** | **Titratable acidity g L^-1^** | **pH** |
| II | 1075 | 12 | 176 ± 3 | 7 ± 1 | 3.38 ± 0.01 |
| III | 1088 | 48 | 186 ± 7 | 7.3 ± 0.3 | 3.37 ± 0.31 |
| IV | 1101 | 39 | 232 ± 8 | 7.4 ± 0.4 | 3.36 ± 0.44 |
| V | 1113 | 2 | 268 ± 7 | 6.8 ± 0.5 | 3.53 ± 0.46 |
| **ND MAR** | | | | | |
| II | 1075 | 17 | 178 ± 4 | 8 ± 1 | 3.19 ± 0.02 |
| III | 1088 | 54 | 227 ± 3 | 8 ± 1 | 3.20 ± 0.02 |
| IV | 1101 | 26 | 253 ± 9 | 8 ± 1 | 3.24 ± 0.02 |
| V | 1113 | 3 | 285 ± 7 | 7 ± 1 | 3.32 ± 0.01 |
| VI | 1126 |  | / | / | / |
| **SY LIM** | | | | | |
| II | 1075 | 12 | 178 ± 3 | 7 ± 1 | 3.40 ± 1.40 |
| III | 1088 | 48 | 197 ± 9 | 5.9 ± 0.2 | 3.61 ± 0.02 |
| IV | 1101 | 39 | 213 ± 9 | 4.9 ± 0.3 | 3.58 ± 0.01 |
| V | 1113 | 2 | / | / | / |
| VI | 1126 | 3 | / | / | / |
| **SY MAR** | | | | | |
| II | 1075 | 6 | 152 ± 4 | 7.1 ± 0.4 | 3.04 ± 0.01 |
| III | 1088 | 24 | 188 ± 5 | 5.2 ± 0.3 | 3.27 ± 0.02 |
| IV | 1101 | 44 | 213 ± 6 | 5.8 ± 0.6 | 3.40 ± 0.03 |
| V | 1113 | 19 | 231 ± 6 | 5.5 ± 0.3 | 3.37 ± 0.01 |
| VI | 1126 | 3 | 279 ± 5 | 4.2 ± 0.3 | 3.49 ± 0.01 |
| **Vintage 2022** | | | | | |
| **ND LIM** | | | | | |
|  | **Density class** | **% m/m** | **Reducing sugars g L^-1^** | **Titratable acidity g L^-1^** | **pH** |
| II | 1075 | 15 | 197 ± 10 | 6.8 ± 0.3 | 3.31 ± 0.01 |
| III | 1088 | 43 | 214 ± 10 | 6.7 ± 0.2 | 3.34 ± 0.01 |
| IV | 1101 | 32 | 235 ± 3 | 6.7 ± 0.2 | 3.34 ± 0.01 |
| V | 1113 | 7 | 257 ± 10 | 6.4 ± 0.3 | 3.46 ± 0.01 |
| VI | 1126 | 7 | 181 ± 6 | 6.0 ± 0.7 | 3.51 ± 0.02 |
| **ND MAR** | | | | | |
| II | 1075 | 22 | 181 ± 8 | 9.1 ± 0.6 | 3.11 ± 0.03 |
| III | 1088 | 43 | 189 ± 4 | 9.2 ± 0.2 | 3.16 ± 0.03 |
| IV | 1101 | 25 | 198 ± 6 | 9.0 ± 0.1 | 3.16 ± 0.01 |
| V | 1113 | 3 | 223 ± 7 | 9.1 ± 0.6 | 3.30 ± 0.03 |
| VI | 1126 | 2 | 253 ± 3 | 7.8 ± 0.6 | 3.35 ± 0.02 |
| **SY LIM** | | | | | |
| II | 1075 | 12 | 140 ± 4 | 6.2 ± 0.5 | 3.33 ± 0.03 |
| III | 1088 | 48 | 171 ± 3 | 6.3 ± 0.3 | 3.44 ± 0.03 |
| IV | 1101 | 39 | 220 ± 7 | 6.4 ± 0.1 | 3.44 ± 0.00 |
| V | 1113 | 2 | 196 ± 6 | 6.0 ± 0.2 | 3.57 ± 0.02 |
| VI | 1126 | 4 | 234 ± 3 | 4.7 ± 0.3 | 3.98 ± 0.02 |
| **SY MAR** | | | | | |
| II | 1075 | 30 | 164 ± 6 | 7.3 ± 0.5 | 3.18 ± 0.01 |
| III | 1088 | 23 | 179 ± 5 | 7.4 ± 0.2 | 3.16 ± 0.01 |
| IV | 1101 | 26 | 205 ± 7 | 6.7 ± 0.8 | 3.24 ± 0.03 |
| V | 1113 | 16 | 230 ± 3 | 6.7 ± 0.3 | 3.29 ± 0.03 |
| VI | 1126 | 3 | 235 ± 3 | 5.1 ± 0.2 | 3.54 ± 0.01 |

Note: Data are reported as mean plus and minus standard error of the mean. ND LIM = *Nero d’Avola* on limestone; ND MAR = *Nero d’Avola* on marlstone; SY LIM = *Syrah* on limestone; SY MAR = *Syrah* on marlstone.

Table S14 Skin and seeds total flavonoids and anthocyanins content during ripening of Nero d’Avola and Syrah cultivar grown on limestone and marlstone soils in 2021 and 2022 vintages.

| **Vintage 2021** | | | | |
| --- | --- | --- | --- | --- |
| **ND LIM** | | | | |
| **Density class** | **% m/m** | **Skins total flavonoids (mg 100 berries^-1^)** | **Seeds total flavonoids (mg 100 berries^-1^)** | **Anthocyanins (mg 100 berries^-1^)** |
| II | **/** | **/** | **/** | **/** |
| III | 12 | 304 ± 22 | 306 ± 4 | 34 ± 6 |
| IV | 48 | 282 ± 60 | 426 ± 123 | 57 ± 1 |
| V | 39 | 454 ± 10 | 520 ± 131 | 111 ± 4 |
| VI | 2 | 289 ± 5 | 340 ± 33 | 91 ± 3 |
| **ND MAR** | | | | |
| **Density class** | **% m/m** | **Skins total flavonoids (mg 100 berries^-1^)** | **Seeds total flavonoids (mg 100 berries^-1^)** | **Anthocyanins (mg 100 berries^-1^)** |
| II | **/** | **/** | **/** | **/** |
| III | 12 | 260 ± 1 | 445 ± 2 | 44 ± 9 |
| IV | 48 | 347 ± 18 | 317 ± 26 | 83 ± 2 |
| V | 39 | 400 ± 60 | 312 ± 8 | 115 ± 3 |
| VI | 2 | 357 ± 37 | 486 ± 10 | 115 ± 7 |
| **SY LIM** | | | | |
| **Density class** | **% m/m** | **Skins total flavonoids (mg 100 berries^-1^)** | **Seeds total flavonoids (mg 100 berries^-1^)** | **Anthocyanins (mg 100 berries^-1^)** |
| II | / | / | / | / |
| III | 12 | 294 ± 41 | 300 ± 24 | 67 ± 13 |
| IV | 48 | 347 ± 6 | 235 ± 6 | 105 ± 3 |
| V | 39 | 404 ± 29 | 263 ± 4 | 126 ± 17 |
| VI | 2 | 367 ± 6 | 198 ± 11 | 109 ± 0 |
| **SY MAR** | | | | |
| **Density class** | **% m/m** | **Skins total flavonoids (mg 100 berries^-1^)** | **Seeds total flavonoids (mg 100 berries^-1^)** | **Anthocyanins (mg 100 berries^-1^)** |
| I | 3 | 248 ± 3 | 282 ± 45 | 39 ± 2 |
| II | 6 | 470 ± 24 | 245 ± 13 | 94 ± 1 |
| III | 24 | 406 ± 9 | 266 ± 12 | 115 ± 6 |
| IV | 44 | 436 ± 55 | 257 ± 9 | 118 ± 1 |
| V | 19 | 583 ± 77 | 229 ± 15 | 213 ± 20 |
| VI | 3 | 536 ± 69 | 237 ± 47 | 185 ± 8 |
|  |  |  |  |  |
| **Vintage 2022** | | | | |
| **ND LIM** | | | | |
| **Density class** | **% m/m** | **Skins total flavonoids (mg 100 berries^-1^)** | **Seeds total flavonoids (mg 100 berries^-1^)** | **Anthocyanins (mg 100 berries^-1^)** |
| I | 3 | 275 ± 8 | 466 ± 49 | 26 ± 2 |
| II | 15 | 327 ± 19 | 495 ± 25 | 54 ± 6 |
| III | 43 | 400 ± 13 | 466 ± 53 | 108 ± 18 |
| IV | 32 | 463 ± 10 | 457 ± 23 | 110 ± 6 |
| V | 7 | 446 ± 1 | 380 ± 15 | 116 ± 1 |
| **ND MAR** | | | | |
| **Density class** | **% m/m** | **Skins total flavonoids (mg 100 berries^-1^)** | **Seeds total flavonoids (mg 100 berries^-1^)** | **Anthocyanins (mg 100 berries^-1^)** |
| I | 7 | 345 ± 17 | 540 ± 2 | 81 ± 8 |
| II | 22 | 340 ± 31 | 487 ± 29 | 76 ± 19 |
| III | 43 | 405 ± 20 | 475 ± 23 | 112 ± 11 |
| IV | 25 | 479 ± 15 | 460 ± 22 | 148 ± 5 |
| V | 3 | 428 ± 1 | 363 ± 6 | 123 ± 8 |
| **SY LIM** | | | | |
| **Density class** | **% m/m** | **Skins total flavonoids (mg 100 berries^-1^)** | **Seeds total flavonoids (mg 100 berries^-1^)** | **Anthocyanins (mg 100 berries^-1^)** |
| I | 6 | 388 ± 31 | 366 ± 6 | 105 ± 1 |
| II | 34 | 384 ± 10 | 418 ± 42 | 117 ± 5 |
| III | 37 | 409 ± 7 | 485 ± 4 | 156 ± 31 |
| IV | 19 | 385 ± 22 | 453 ± 24 | 137 ± 8 |
| V | 3 | 489 ± 6 | 448 ± 11 | 197 ± 17 |
| VI | 1 | 503 ± 4 | 416 ± 8 | 178 ± 8 |
| **SY MAR** | | | | |
| **Density class** | **% m/m** | **Skins total flavonoids (mg 100 berries^-1^)** | **Seeds total flavonoids (mg 100 berries^-1^)** | **Anthocyanins (mg 100 berries^-1^)** |
| I | 4 | 327 ± 18 | 515 ± 22 | 110 ± 1 |
| II | 30 | 351 ± 10 | 544 ± 2 | 108 ± 12 |
| III | 23 | 504 ± 46 | 473 ± 9 | 150 ± 18 |
| IV | 26 | 507 ± 31 | 500 ± 33 | 184 ± 8 |
| V | 16 | 550 ± 29 | 340 ± 36 | 215 ± 19 |
| VI | 1 | 439 ± 10 | 387 ± 10 | 174 ± 2 |

Note: Data are reported as mean plus and minus standard error of the mean. ND LIM = *Nero d’Avola* on limestone; ND MAR = *Nero d’Avola* on marlstone; SY LIM = *Syrah* on limestone; SY MAR = *Syrah* on marlstone. Density class: I= 1071, II = 1075, III = 1088, IV = 1101, V = 1113, VI = 1126 kg m^-3^.

Table S15 Flavonols content (g kg^-1^ of berries) during ripening of Nero d’Avola and Syrah cultivar grown on limestone and marlstone soils in 2021 and 2022 vintages.

| **Vintage 2021** | | | | | | | | | | | | |
| --- | --- | --- | --- | --- | --- | --- | --- | --- | --- | --- | --- | --- |
| **ND LIM** | | | | | | | | | | | | |
| **Density class** | **% m/m** | **Myricetin 3-glucuronide** | **Myricetin 3-glucoside** | **Quercetin 3-glucuronide** | **Quercetin 3-glucoside** | **Isorhamnetin 3-glucoside** | **Laricitrin 3-glucoside** | **kaempferol 3-glucuronide** | **kaempferol 3-glucoside** | **Myricetin aglycon** | **Syringetin 3-glucoside** | **Quercetin aglycon** |
| I | 12 | 0.00 ± 0.00 | 8.38 ± 0.11 | 68.20 ± 1.38 | 45.80 ± 8.55 | 67.41 ± 7.72 | 0.00 ± 0.00 | 2.58 ± 0.01 | 22.52 ± 2.88 | 0.50 ± 0.02 | 0.27 ± 0.27 | 27.75 ± 0.88 |
| II | 48 | 2.77 ± 0.08 | 28.77 ± 2.02 | 12.55 ± 1.43 | 11.80 ± 1.12 | 6.33 ± 0.25 | 0.00 ± 0.00 | 0.80 ± 0.06 | 1.05 ± 0.14 | 3.97 ± 0.14 | 0.00 ± 0.00 | 9.22 ± 1.00 |
| III | 39 | 2.61 ± 0.31 | 31.77 ± 4.48 | 9.63 ± 1.18 | 10.55 ± 1.24 | 7.70 ± 0.85 | 0.00 ± 0.00 | 0.89 ± 0.18 | 0.99 ± 0.05 | 4.67 ± 0.36 | 0.00 ± 0.00 | 12.32 ± 1.71 |
| IV | 2 | 2.98 ± 0.14 | 42.30 ± 6.09 | 19.02 ± 6.28 | 20.78 ± 6.43 | 8.71 ± 1.49 | 0.00 ± 0.00 | 0.96 ± 0.39 | 1.74 ± 0.48 | 6.77 ± 0.66 | 0.00 ± 0.00 | 15.14 ± 0.59 |
| **ND MAR** | | | | | | | | | | | | |
| **Density class** | **% m/m** | **Myricetin 3-glucuronide** | **Myricetin 3-glucoside** | **Quercetin 3-glucuronide** | **Quercetin 3-glucoside** | **Isorhamnetin 3-glucoside** | **Laricitrin 3-glucoside** | **kaempferol 3-glucuronide** | **kaempferol 3-glucoside** | **Myricetin aglycon** | **Syringetin 3-glucoside** | **Quercetin aglycon** |
| I | 17 | 14.17 ± 1.92 | 115.78 ± 14.76 | 75.84 ± 8.42 | 74.36 ± 12.63 | 0.00 ± 0.00 | 0.00 ± 0.00 | 3.66 ± 0.58 | 23.34 ± 4.51 | 0.25 ± 0.25 | 0.00 ± 0.00 | 42.28 ± 3.02 |
| II | 54 | 14.22 ± 0.00 | 120.28 ± 0.11 | 73.31 ± 1.38 | 82.74 ± 8.55 | 0.00 ± 7.72 | 0.00 ± 0.00 | 4.06 ± 0.01 | 26.02 ± 2.88 | 0.93 ± 0.02 | 0.00 ± 0.27 | 43.04 ± 0.88 |
| III | 54 | 13.68 ± 0.08 | 118.03 ± 2.02 | 52.61 ± 1.43 | 64.30 ± 1.12 | 0.00 ± 0.25 | 0.00 ± 0.00 | 4.12 ± 0.06 | 24.75 ± 0.14 | 1.21 ± 0.14 | 0.00 ± 0.00 | 54.10 ± 1.00 |
| IV | 26 | 4.14 ± 0.31 | 44.92 ± 4.48 | 15.72 ± 1.18 | 9.97 ± 1.24 | 8.37 ± 0.85 | 0.00 ± 0.00 | 2.09 ± 0.18 | 6.88 ± 0.05 | 0.27 ± 0.36 | 0.00 ± 0.00 | 19.21 ± 1.71 |
| V | 3 | 4.55 ± 0.14 | 56.64 ± 6.09 | 17.23 ± 6.28 | 14.11 ± 6.43 | 9.88 ± 1.49 | 0.00 ± 0.00 | 2.14 ± 0.39 | 8.31 ± 0.48 | 0.53 ± 0.66 | 0.00 ± 0.00 | 19.78 ± 0.59 |
| **SY LIM** | | | | | | | | | | | | |
| **Density class** | **% m/m** | **Myricetin 3-glucuronide** | **Myricetin 3-glucoside** | **Quercetin 3-glucuronide** | **Quercetin 3-glucoside** | **Isorhamnetin 3-glucoside** | **Laricitrin 3-glucoside** | **kaempferol 3-glucuronide** | **kaempferol 3-glucoside** | **Myricetin aglycon** | **Syringetin 3-glucoside** | **Quercetin aglycon** |
| I | 12 | 15.08 ± 2.37 | 164.03 ± 32.28 | 111.81 ± 22.99 | 326.33 ± 56.61 | 0.00 ± 0.00 | 0.00 ± 0.00 | 0.00 ± 0.00 | 151.35 ± 21.74 | 2.37 ± 0.45 | 0.00 ± 0.00 | 37.56 ± 6.94 |
| II | 48 | 22.26 ± 2.32 | 226.95 ± 13.34 | 88.57 ± 8.16 | 255.68 ± 18.25 | 1.36 ± 1.36 | 0.00 ± 0.00 | 0.00 ± 0.00 | 152.34 ± 13.19 | 1.52 ± 0.31 | 0.00 ± 0.00 | 46.92 ± 0.01 |
| III | 39 | 4.38 ± 0.38 | 53.97 ± 1.97 | 22.20 ± 2.01 | 62.31 ± 1.43 | 0.36 ± 0.36 | 0.00 ± 0.00 | 0.00 ± 0.00 | 37.77 ± 2.33 | 0.42 ± 0.19 | 0.00 ± 0.00 | 12.20 ± 0.91 |
| IV | 2 | 4.91 ± 0.08 | 62.61 ± 3.36 | 21.55 ± 1.45 | 36.21 ± 2.61 | 24.03 ± 0.72 | 0.00 ± 0.00 | 0.00 ± 0.00 | 44.48 ± 1.92 | 0.00 ± 0.00 | 0.00 ± 0.00 | 14.95 ± 1.49 |
| **SY MAR** | | | | | | | | | | | | |
| **Density class** | **% m/m** | **Myricetin 3-glucuronide** | **Myricetin 3-glucoside** | **Quercetin 3-glucuronide** | **Quercetin 3-glucoside** | **Isorhamnetin 3-glucoside** | **Laricitrin 3-glucoside** | **kaempferol 3-glucuronide** | **kaempferol 3-glucoside** | **Myricetin aglycon** | **Syringetin 3-glucoside** | **Quercetin aglycon** |
| I | 3 | 8.78 ± 0.33 | 63.97 ± 3.79 | 137.26 ± 5.86 | 182.70 ± 16.94 | 0.00 ± 0.00 | 0.00 ± 0.00 | 0.00 ± 0.00 | 60.52 ± 6.33 | 1.16 ± 0.03 | 0.00 ± 0.00 | 21.55 ± 2.50 |
| II | 6 | 10.25 ± 0.51 | 99.64 ± 10.73 | 100.38 ± 13.37 | 213.17 ± 39.01 | 0.00 ± 0.00 | 0.00 ± 0.00 | 0.00 ± 0.00 | 82.19 ± 14.47 | 1.64 ± 0.13 | 0.00 ± 0.00 | 27.59 ± 0.48 |
| III | 24 | 11.61 ± 1.09 | 121.87 ± 4.27 | 128.94 ± 2.85 | 266.26 ± 4.77 | 0.00 ± 0.00 | 0.00 ± 0.00 | 0.00 ± 0.00 | 113.37 ± 4.46 | 0.96 ± 0.96 | 0.00 ± 0.00 | 29.87 ± 9.83 |
| IV | 44 | 3.90 ± 0.19 | 39.10 ± 2.67 | 44.61 ± 6.99 | 93.49 ± 12.07 | 0.00 ± 0.00 | 0.00 ± 0.00 | 0.00 ± 0.00 | 38.14 ± 4.58 | 0.50 ± 0.07 | 0.00 ± 0.00 | 6.81 ± 3.81 |
| V | 19 | 7.02 ± 4.06 | 72.47 ± 26.47 | 48.36 ± 24.30 | 89.59 ± 63.32 | 4.64 ± 4.64 | 0.00 ± 0.00 | 0.00 ± 0.00 | 33.88 ± 21.84 | 0.56 ± 0.56 | 0.00 ± 0.00 | 19.20 ± 5.37 |
| VI | 3 | 9.35 ± 0.30 | 72.90 ± 6.89 | 63.58 ± 4.61 | 122.37 ± 10.53 | 7.06 ± 7.06 | 0.00 ± 0.00 | 0.00 ± 0.00 | 46.28 ± 3.84 | 0.67 ± 0.11 | 0.00 ± 0.00 | 15.80 ± 2.77 |
| **Vintage 2022** | | | | | | | | | | | | |
| **ND LIM** | | | | | | | | | | | | |
| **Density class** | **% m/m** | **Myricetin 3-glucuronide** | **Myricetin 3-glucoside** | **Quercetin 3-glucuronide** | **Quercetin 3-glucoside** | **Isorhamnetin 3-glucoside** | **Laricitrin 3-glucoside** | **kaempferol 3-glucuronide** | **kaempferol 3-glucoside** | **Myricetin aglycon** | **Syringetin 3-glucoside** | **Quercetin aglycon** |
| I | 3 | 6.22 ± 0.18 | 70.33 ± 0.47 | 84.09 ± 2.86 | 42.53 ± 1.15 | 14.49 ± 0.47 | 3.23 ± 0.17 | 2.53 ± 0.16 | 11.31 ± 0.50 | 1.43 ± 0.23 | 1.00 ± 0.05 | 0.00 ± 0.00 |
| II | 12 | 2.07 ± 0.36 | 27.30 ± 2.66 | 13.85 ± 0.29 | 10.04 ± 0.20 | 5.18 ± 0.32 | 1.10 ± 0.18 | 0.56 ± 0.01 | 3.59 ± 0.15 | 0.29 ± 0.02 | 0.12 ± 0.12 | 0.00 ± 0.00 |
| III | 43 | 2.03 ± 0.79 | 31.70 ± 9.19 | 12.11 ± 4.19 | 7.55 ± 2.87 | 6.10 ± 1.76 | 1.00 ± 0.74 | 0.37 ± 0.20 | 4.26 ± 0.92 | 0.18 ± 0.18 | 0.20 ± 0.20 | 0.00 ± 0.00 |
| IV | 39 | 3.00 ± 0.05 | 43.07 ± 1.89 | 15.35 ± 0.43 | 10.95 ± 0.04 | 8.17 ± 1.24 | 1.61 ± 0.03 | 3.66 ± 3.11 | 3.33 ± 3.10 | 0.34 ± 0.11 | 0.14 ± 0.14 | 0.00 ± 0.00 |
| V | 2 | 4.50 ± 0.57 | 63.65 ± 7.60 | 19.75 ± 1.34 | 16.99 ± 0.29 | 13.27 ± 0.50 | 2.59 ± 0.17 | 1.02 ± 0.12 | 9.35 ± 0.09 | 0.53 ± 0.06 | 0.17 ± 0.17 | 0.00 ± 0.00 |
| **ND MAR** | | | | | | | | | | | | |
| **Density class** | **% m/m** | **Myricetin 3-glucuronide** | **Myricetin 3-glucoside** | **Quercetin 3-glucuronide** | **Quercetin 3-glucoside** | **Isorhamnetin 3-glucoside** | **Laricitrin 3-glucoside** | **kaempferol 3-glucuronide** | **kaempferol 3-glucoside** | **Myricetin aglycon** | **Syringetin 3-glucoside** | **Quercetin aglycon** |
| I | 7 | 10.46 ± 10.46 | 95.94 ± 95.94 | 92.30 ± 10.62 | 92.94 ± 10.24 | 16.86 ± 15.75 | 6.72 ± 0.86 | 24.24 ± 14.39 | 9.28 ± 7.59 | 0.71 ± 0.02 | 0.00 ± 0.00 | 0.00 ± 0.00 |
| II | 17 | 20.42 ± 1.28 | 195.48 ± 1.18 | 88.53 ± 19.90 | 68.34 ± 4.10 | 28.23 ± 2.77 | 10.37 ± 1.14 | 6.71 ± 0.47 | 19.98 ± 2.15 | 1.82 ± 0.22 | 1.74 ± 0.78 | 0.00 ± 0.00 |
| III | 43 | 4.89 ± 0.73 | 48.43 ± 2.37 | 26.86 ± 1.34 | 24.64 ± 4.41 | 8.61 ± 0.18 | 1.77 ± 0.27 | 6.13 ± 3.39 | 2.69 ± 2.10 | 0.33 ± 0.15 | 0.00 ± 0.00 | 0.00 ± 0.00 |
| IV | 26 | 7.64 ± 0.02 | 72.72 ± 0.63 | 30.81 ± 1.49 | 28.29 ± 0.54 | 10.96 ± 0.15 | 3.20 ± 0.32 | 4.09 ± 0.29 | 5.91 ± 0.07 | 0.33 ± 0.05 | 0.15 ± 0.15 | 0.00 ± 0.00 |
| V | 3 | 6.89 ± 0.35 | 71.56 ± 0.89 | 53.33 ± 4.71 | 58.12 ± 14.20 | 12.94 ± 2.82 | 2.31 ± 0.35 | 4.65 ± 0.32 | 15.79 ± 10.70 | 0.35 ± 0.01 | 0.38 ± 0.38 | 0.00 ± 0.00 |
| **SY LIM** | | | | | | | | | | | | |
| **Density class** | **% m/m** | **Myricetin 3-glucuronide** | **Myricetin 3-glucoside** | **Quercetin 3-glucuronide** | **Quercetin 3-glucoside** | **Isorhamnetin 3-glucoside** | **Laricitrin 3-glucoside** | **kaempferol 3-glucuronide** | **kaempferol 3-glucoside** | **Myricetin aglycon** | **Syringetin 3-glucoside** | **Quercetin aglycon** |
| I | 6 | 13.98 ± 0.87 | 133.85 ± 9.51 | 152.33 ± 5.42 | 226.81 ± 17.28 | 45.83 ± 3.26 | 4.43 ± 0.15 | 8.77 ± 0.23 | 111.02 ± 9.10 | 1.73 ± 0.36 | 4.29 ± 0.49 | 0.00 ± 0.00 |
| II | 12 | 19.86 ± 2.97 | 174.77 ± 16.19 | 159.77 ± 27.16 | 300.63 ± 50.57 | 63.62 ± 10.73 | 7.98 ± 1.35 | 11.99 ± 3.05 | 140.07 ± 15.93 | 1.44 ± 0.60 | 4.23 ± 1.41 | 0.00 ± 0.00 |
| III | 42 | 4.11 ± 0.85 | 39.72 ± 4.54 | 32.47 ± 5.63 | 59.10 ± 7.90 | 13.94 ± 1.84 | 1.36 ± 0.36 | 2.66 ± 0.67 | 29.25 ± 3.28 | 0.48 ± 0.28 | 0.72 ± 0.08 | 0.00 ± 0.00 |
| IV | 39 | 4.91 ± 0.23 | 53.56 ± 1.38 | 33.85 ± 3.59 | 67.69 ± 3.47 | 19.61 ± 0.47 | 1.78 ± 0.11 | 3.25 ± 0.11 | 37.07 ± 0.31 | 0.28 ± 0.02 | 0.90 ± 0.07 | 0.00 ± 0.00 |
| V | 2 | 4.20 ± 0.71 | 55.44 ± 4.47 | 30.19 ± 7.67 | 59.37 ± 13.25 | 20.48 ± 1.24 | 1.68 ± 0.34 | 3.06 ± 0.70 | 39.20 ± 3.23 | 0.39 ± 0.07 | 1.02 ± 0.18 | 0.00 ± 0.00 |
| **SY MAR** | | | | | | | | | | | | |
| **Density class** | **% m/m** | **Myricetin 3-glucuronide** | **Myricetin 3-glucoside** | **Quercetin 3-glucuronide** | **Quercetin 3-glucoside** | **Isorhamnetin 3-glucoside** | **Laricitrin 3-glucoside** | **kaempferol 3-glucuronide** | **kaempferol 3-glucoside** | **Myricetin aglycon** | **Syringetin 3-glucoside** | **Quercetin aglycon** |
| I | 4 | 2.78 ± 0.34 | 21.14 ± 3.05 | 45.47 ± 3.45 | 28.03 ± 0.51 | 7.10 ± 1.26 | 0.00 ± 0.00 | 1.92 ± 0.37 | 11.55 ± 0.08 | 0.40 ± 0.19 | 0.19 ± 0.19 | 0.00 ± 0.00 |
| II | 30 | 6.05 ± 0.38 | 67.60 ± 0.05 | 61.22 ± 8.28 | 101.34 ± 12.51 | 22.87 ± 1.18 | 1.95 ± 0.10 | 2.84 ± 1.01 | 50.49 ± 2.03 | 0.91 ± 0.16 | 1.31 ± 0.14 | 0.00 ± 0.00 |
| III | 23 | 2.12 ± 0.70 | 26.59 ± 8.08 | 22.75 ± 4.89 | 37.83 ± 12.79 | 8.81 ± 2.58 | 0.36 ± 0.36 | 1.52 ± 0.21 | 17.69 ± 6.93 | 0.23 ± 0.11 | 0.28 ± 0.28 | 0.00 ± 0.00 |
| IV | 26 | 3.46 ± 0.21 | 39.44 ± 2.43 | 26.13 ± 1.72 | 36.25 ± 2.16 | 9.95 ± 0.99 | 1.56 ± 0.06 | 1.84 ± 0.14 | 15.79 ± 1.01 | 0.23 ± 0.02 | 0.50 ± 0.05 | 0.00 ± 0.00 |
| V | 16 | 3.69 ± 0.00 | 50.55 ± 4.27 | 20.91 ± 3.66 | 46.34 ± 8.74 | 18.10 ± 0.49 | 1.56 ± 0.05 | 1.55 ± 0.28 | 32.61 ± 1.67 | 0.30 ± 0.02 | 0.85 ± 0.10 | 0.00 ± 0.00 |
| VI | 1 | 3.99 ± 0.00 | 55.01 ± 0.00 | 38.09 ± 0.00 | 71.16 ± 0.00 | 17.92 ± 0.00 | 1.24 ± 0.00 | 2.60 ± 0.00 | 30.01 ± 0.00 | 0.32 ± 0.00 | 0.99 ± 0.00 | 0.00 ± 0.00 |

Note: Data are reported as mean plus and minus standard error of the mean. ND LIM = *Nero d’Avola* on limestone; ND MAR = *Nero d’Avola* on marlstone; SY LIM = *Syrah* on limestone; SY MAR = *Syrah* on marlstone. Density class: I= 1071, II = 1075, III = 1088, IV = 1101, V = 1113, VI = 1126 kg m^-3^.

Table S16 Hydrocycinnamoyltartaric acids (HCTAs) content (g kg^-1^ of berries) during ripening of Nero d’Avola and Syrah cultivar grown on limestone and marlstone soils in 2021 and 2022 vintages.

| **Vintage 2021** | | | | | |
| --- | --- | --- | --- | --- | --- |
| **ND LIM** | | | | | |
|  | **Density class** | **% m/m** | **Caftaric acid** | **Coutaric acid** | **Feftaric acid** |
| II | 1075 | 12 | 785 ± 31 | 49 ± 8 | 195 ± 23 |
| III | 1088 | 48 | 238 ± 18 | 35 ± 19 | 47 ± 4 |
| IV | 1101 | 39 | 144 ± 13 | 23 ± 12 | 32 ± 12 |
| V | 1113 | 2 | 241 ± 19 | 12 ± 1 | 58 ± 8 |
| **ND MAR** | | | | | |
| II | 1075 | 17 | 1,002 ± 75 | 53 ± 3 | 274 ± 1 |
| III | 1088 | 54 | 922 ± 62 | 42 ± 9 | 239 ± 22 |
| IV | 1101 | 26 | 194 ± 6 | 17 ± 0 | 64 ± 2 |
| V | 1113 | 3 | 268 ± 5 | 20 ± 0 | 73 ± 2 |
| **SY LIM** | | | | | |
| II | 1075 | 12 | 516 ± 58 | 75 ± 4 | 592 ± 56 |
| III | 1088 | 48 | 544 ± 14 | 75 ± 7 | 552 ± 15 |
| IV | 1101 | 39 | 118 ± 26 | 16 ± 3 | 118 ± 21 |
| V | 1113 | 2 | 108 ± 4 | 19 ± 0 | 139 ± 10 |
| **SY MAR** | | | | | |
| II | 1075 | 6 | 527 ± 32 | 99 ± 5 | 642 ± 34 |
| III | 1088 | 24 | 509 ± 16 | 166 ± 79 | 449 ± 227 |
| IV | 1101 | 44 | 129 ± 2 | 26 ± 1 | 159 ± 5 |
| V | 1113 | 19 | 185 ± 17 | 33 ± 1 | 247 ± 15 |
| VI | 1126 | 3 | 136 ± 13 | 26 ± 1 | 178 ± 11 |
| **Vintage 2022** | | | | | |
| **ND LIM** | | | | | |
| II | 1075 | 15 | 159 ± 13 | 38 ± 2 | 7.1 ± 0.3 |
| III | 1088 | 43 | 140 ± 12 | 33 ± 3 | 11 ± 2 |
| IV | 1101 | 32 | 160 ± 3 | 37 ± 1 | 9.9 ± 0.4 |
| VI | 1113 | 7 | 212 ± 15 | 44 ± 0 | 14 ± 2 |
| **ND MAR** | | | | | |
| II | 1075 | 22 | 1,141 ± 57 | 154 ± 109 | 166 ± 141 |
| III | 1088 | 43 | 240 ± 13 | 76 ± 11 | 7 ± 1 |
| IV | 1101 | 25 | 258 ± 5 | 61 ± 2 | 10 ± 1 |
| V | 1113 | 3 | 220 ± 5 | 107 ± 44 | 11 ± 1 |
| **SY LIM** | | | | | |
| II | 1075 | 12 | 109 ± 8 | 108 ± 7 | 7 ± 2 |
| III | 1088 | 48 | 111 ± 4 | 100 ± 1 | 15 ± 1 |
| IV | 1101 | 39 | 97 ± 4 | 97 ± 8 | 26 ± 0 |
| V | 1113 | 2 | 0 ± 3 | 0 ± 3 | 0 ± 4 |
| **SY MAR** | | | | | |
| II | 1075 | 30 | 368 ± 14 | 436 ± 0 | 41 ± 2 |
| III | 1088 | 23 | 126 ± 21 | 142 ± 23 | 13 ± 1 |
| IV | 1101 | 26 | 137 ± 8 | 86 ± 5 | 12 ± 1 |
| V | 1113 | 16 | 109 ± 27 | 122 ± 30 | 15 ± 1 |

Note: Data are reported as mean plus and minus standard error of the mean. ND LIM = *Nero d’Avola* on limestone; ND MAR = *Nero d’Avola* on marlstone; SY LIM = *Syrah* on limestone; SY MAR = *Syrah* on marlstone.

Table S17 Coefficient variation of some technological parameters and phenolic compounds

| **Factor** | **CV**  **Reducing sugars** | **CV**  **Titratable**  **acidity** | **CV**  **pH** | **CV_Caftaric acid** | **CV_Coutaric acid** | **CV_Feftaric acid** | **CV_Quercetin 3-glucoside** | **CV**  **NAF skin** | **CV**  **NAF seeds** | **CV**  **Anthocyanins** |
| --- | --- | --- | --- | --- | --- | --- | --- | --- | --- | --- |
| Cultivar (n=4) |  |  |  |  |  |  |  |  |  |  |
| CH | 0.14 ± 0.04 | 0.07 ± 0.03 ab | 0.011 ± 0.001 | 0.86 ± 0.21 | 0.89 ± 0.19 | 0.95 ± 0.13 | 0.73 ± 0.17 | 0.12 ± 0.02 | 0.18 ± 0.02 | 0.00 ± 0.00 b |
| GR | 0.13 ± 0.02 | 0.06 ± 0.01 bc | 0.021 ± 0.001 | 0.52 ± 0.12 | 0.52 ± 0.11 | 0.68 ± 0.23 | 0.62 ± 0.15 | 0.12 ± 0.01 | 0.14 ± 0.02 | 0.00 ± 0.00 b |
| ND | 0.10 ± 0.01 | 0.021 ± 0.001 c | 0.02 ± 0.01 | 0.66 ± 0.07 | 0.59 ± 0.07 | 0.93 ± 0.41 | 0.62 ± 0.05 | 0.16 ± 0.03 | 0.20 ± 0.06 | 0.25 ± 0.03 a |
| SY | 0.11 ± 0.02 | 0.111 ± 0.001 a | 0.03 ± 0.01 | 0.64 ± 0.04 | 0.78 ± 0.10 | 0.65 ± 0.04 | 0.63 ± 0.07 | 0.17 ± 0.03 | 0.15 ± 0.07 | 0.28 ± 0.06 a |
| Sign. | ns | ** | ns | ns | ns | ns | ns | ns | ns | *** |
| Year (n=8) |  |  |  |  |  |  |  |  |  |  |
| 2021 | 0.13 ± 0.02 | 0.07 ± 0.02 | 0.021 ± 0.001 | 0.70 ± 0.12 | 0.72 ± 0.13 | 0.73 ± 0.12 | 0.70 ± 0.10 | 0.14 ± 0.02 | 0.21 ± 0.04 | 0.14 ± 0.06 |
| 2022 | 0.11 ± 0.01 | 0.06 ± 0.01 | 0.021 ± 0.001 | 0.64 ± 0.06 | 0.67 ± 0.05 | 0.87 ± 0.20 | 0.60 ± 0.04 | 0.14 ± 0.02 | 0.13 ± 0.01 | 0.12 ± 0.05 |
| Sign. | ns | ns | ns | ns | ns | ns | ns | ns | ns | ns |
| Soil (n=8) |  |  |  |  |  |  |  |  |  |  |
| LIM | 0.14 ± 0.02 | 0.08 ± 0.02 | 0.02 ± 0.00 | 0.75 ± 0.07 | 0.73 ± 0.07 | 0.79 ± 0.11 | 0.76 ± 0.09 | 0.14 ± 0.01 | 0.14 ± 0.03 | 0.11 ± 0.04 |
| MAR | 0.10 ± 0.01 | 0.06 ± 0.01 | 0.02 ± 0.00 | 0.59 ± 0.11 | 0.66 ± 0.12 | 0.81 ± 0.21 | 0.54 ± 0.05 | 0.14 ± 0.02 | 0.19 ± 0.03 | 0.15 ± 0.06 |
| Sign. | * | . | ns | ns | ns | ns | ns | ns | ns | * |
| Cultivar:Soil. (n=2) |  |  |  |  |  |  |  |  |  |  |
| CH:LIM | 0.20 ± 0.04 a | 0.11 ± 0.05 ab | 0.01 ± 0.01 | 1.01 ± 0.22 | 0.99 ± 0.26 | 0.91 ± 0.42 | 0.85 ± 0.52 | 0.15 ± 0.05 | 0.15 ± 0.01 | 0.00 ± 0.00 c |
| GR:LIM | 0.16 ± 0.03 ab | 0.07 ± 0.01 abc | 0.02 ± 0.01 | 0.661 ± 0.001 | 0.63 ± 0.02 | 1.02 ± 0.34 | 0.79 ± 0.23 | 0.11 ± 0.00 | 0.15 ± 0.02 | 0.00 ± 0.00 c |
| ND:LIM | 0.10 ± 0.02 ab | 0.01 ± 0.01 c | 0.01 ± 0.00 | 0.68 ± 0.08 | 0.63 ± 0.10 | 0.59 ± 0.41 | 0.68 ± 0.10 | 0.19 ± 0.01 | 0.21 ± 0.13 | 0.27 ± 0.01 ab |
| SY:LIM | 0.08 ± 0.03 b | 0.11 ± 0.01 ab | 0.03 ± 0.01 | 0.64 ± 0.11 | 0.68 ± 0.15 | 0.66 ± 0.12 | 0.71 ± 0.19 | 0.12 ± 0.03 | 0.08 ± 0.07 | 0.18 ± 0.03 b |
| CH:MAR | 0.08 ± 0.03 b | 0.04 ± 0.02 bc | 0.01 ± 0.001 | 0.71 ± 0.64 | 0.79 ± 0.59 | 0.99 ± 0.14 | 0.62 ± 0.18 | 0.09 ± 0.02 | 0.21 ± 0.03 | 0.00 ± 0.00 c |
| GR:MAR | 0.111 ± 0.001 ab | 0.04 ± 0.02 | 0.02 ± 0.001 | 0.4 ± 0.3 | 0.40 ± 0.31 | 0.33 ± 0.19 | 0.44 ± 0.29 | 0.13 ± 0.01 | 0.13 ± 0.05 | 0.00 ± 0.00 c |
| ND:MAR | 0.09 ± 0.02 ab | 0.03 ± 0.01 c | 0.03 ± 0.01 | 0.63 ± 0.23 | 0.56 ± 0.22 | 1.26 ± 1.18 | 0.56 ± 0.04 | 0.12 ± 0.08 | 0.20 ± 0.15 | 0.24 ± 0.08 b |
| SY:MAR | 0.13 ± 0.02 ab | 0.12 ± 0.01 a | 0.03 ± 0.02 | 0.63 ± 0.06 | 0.89 ± 0.24 | 0.64 ± 0.06 | 0.55 ± 0.03 | 0.22 ± 0.00 | 0.23 ± 0.16 | 0.37 ± 0.03 a |
| Sign. | * | * | ns | ns | ns | ns | ns | * | ns | ** |

Note: Data are reported as mean plus and minus standard error of the mean. Sign. =ANOVA. Statistical significance is indicated for each factor and their interaction (ns = not significant, . = 90% of significance, * = 95% of significance, ** = 99% of significance, *** = 99.9% of significance). Different letters indicate statistically significant differences according to post-hoc tests (*p* < 0.05). CH LIM = *Chardonnay* on limestone; CH MAR = *Chardonnay* on marlstone; GR LIM = *Grillo* on limestone; GR MAR = *Grillo* on marlstone; ND LIM = *Nero d’Avola* on limestone; ND MAR = *Nero d’Avola* on marlstone; SY LIM = *Syrah* on limestone; SY MAR = *Syrah* on marlstone.


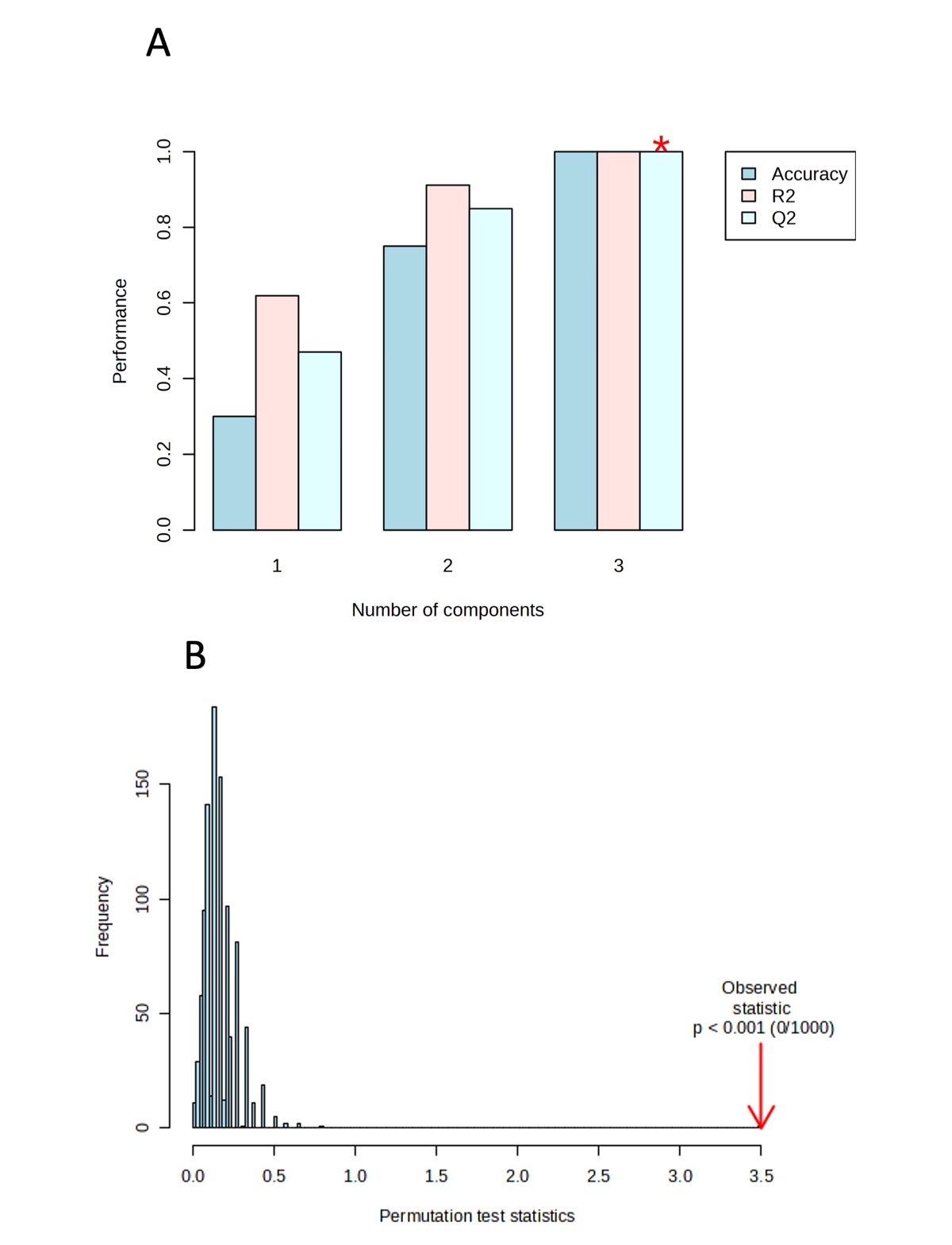


Figure S5 Permutation test (B) and cross validation (A) (n = 1000) for the PLS-DA analysis carried out on white grapes (Chardonnay and Grillo) composition.


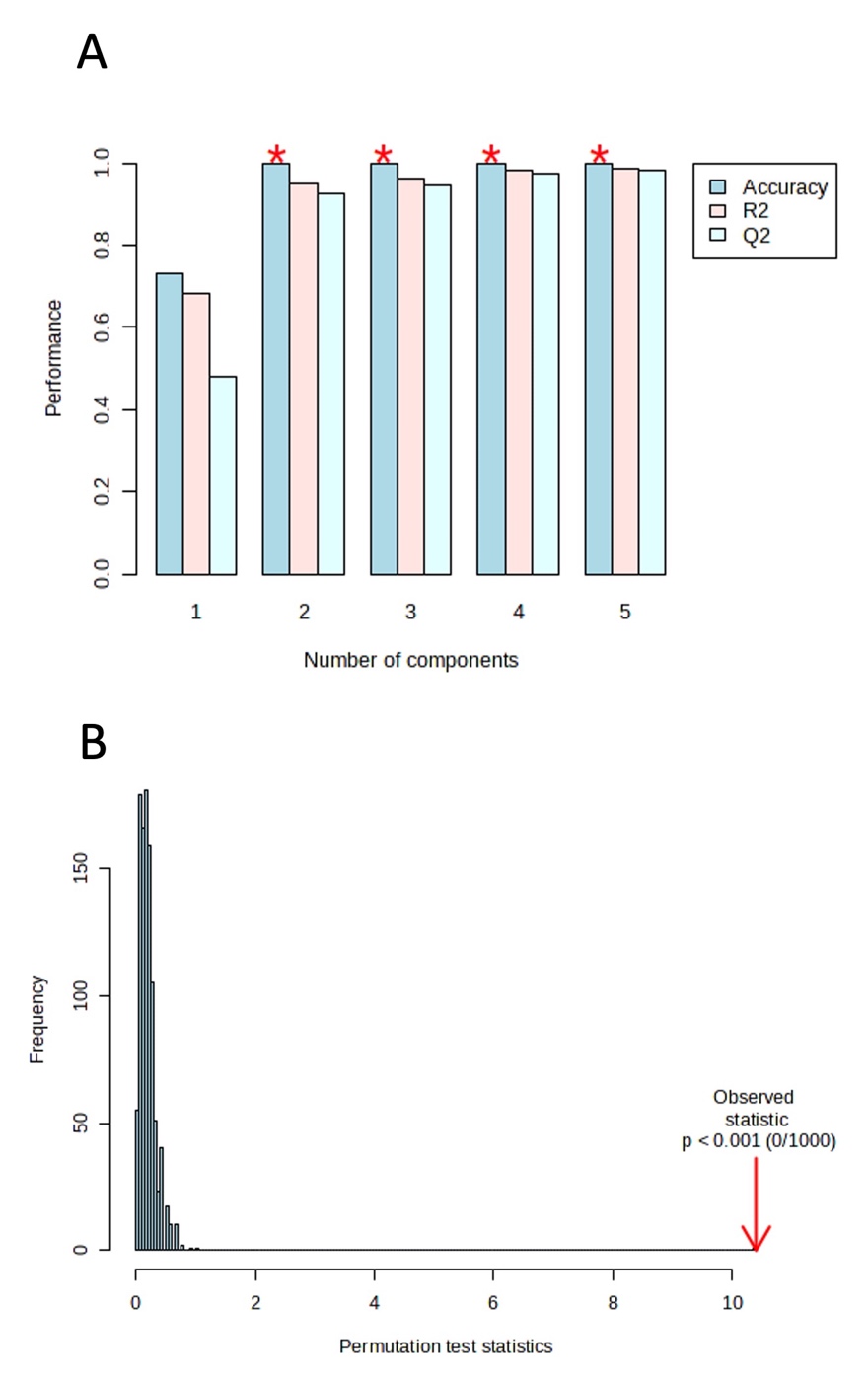


Figure S6 Permutation test (B) and cross validation (A) (n = 1000) for the PLS-DA analysis carried out on red grapes (Nero d’Avola and Syrah) composition.
